# Metabolic balance in colorectal cancer is maintained by optimal Wnt signaling levels

**DOI:** 10.15252/msb.202110874

**Published:** 2022-07-29

**Authors:** Katharina Imkeller, Giulia Ambrosi, Nancy Klemm, Ainara Claveras Cabezudo, Luisa Henkel, Wolfgang Huber, Michael Boutros

**Affiliations:** ^1^ German Cancer Research Center (DKFZ) Division Signaling and Functional Genomics and Heidelberg University Heidelberg Germany; ^2^ European Molecular Biology Laboratory Heidelberg Germany; ^3^ Brandenburg University of Technology Cottbus‐Senftenberg Germany; ^4^ Heidelberg University Heidelberg Germany; ^5^ Present address: Edinger Institute (Neurological Institute), University Hospital Frankfurt am Main, Frankfurt Cancer Institute University Cancer Center Frankfurt am Main Frankfurt am Main Germany

**Keywords:** APC, functional genomics, multi‐omic data integration, quantitative signaling, synthetic lethality, Cancer, Signal Transduction

## Abstract

Wnt pathways are important for the modulation of tissue homeostasis, and their deregulation is linked to cancer development. Canonical Wnt signaling is hyperactivated in many human colorectal cancers due to genetic alterations of the negative Wnt regulator *APC*. However, the expression levels of Wnt‐dependent targets vary between tumors, and the mechanisms of carcinogenesis concomitant with this Wnt signaling dosage have not been understood. In this study, we integrate whole‐genome CRISPR/Cas9 screens with large‐scale multi‐omic data to delineate functional subtypes of cancer. We engineer *APC* loss‐of‐function mutations and thereby hyperactivate Wnt signaling in cells with low endogenous Wnt activity and find that the resulting engineered cells have an unfavorable metabolic equilibrium compared with cells which naturally acquired Wnt hyperactivation. We show that the dosage level of oncogenic Wnt hyperactivation impacts the metabolic equilibrium and the mitochondrial phenotype of a given cell type in a context‐dependent manner. These findings illustrate the impact of context‐dependent genetic interactions on cellular phenotypes of a central cancer driver mutation and expand our understanding of quantitative modulation of oncogenic signaling in tumorigenesis.

## Graphical abstract

Whole‐genome CRISPR screens in genome‐engineered colorectal cancer (CRC) cell lines combined with large‐scale multi‐omic data integration reveal the role of diverging patterns of Wnt hyperactivation during the development of distinct CRC subtypes.

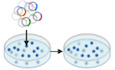

## Introduction

Colorectal cancer (CRC) is among the most common types of cancer worldwide, and the patients' survival rates remain poor, especially for advanced stages (Bray *et al*, 2018). Major effort has been undertaken to explore the heterogeneity of this disease to understand the mechanisms of tumorigenesis and identify personalized treatment strategies. The classification of tumors into consensus molecular subtypes (CMS; Guinney *et al*, 2015; Dienstmann *et al*, 2017) has been an important step toward this goal. The CMS classification is based on transcriptome profiling and has been enabled by genome and transcriptome profiling studies of large patient cohorts (Cancer Genome Atlas Network, 2012). The four CMSs differ in their mutational profile, their infiltration by immune and stromal cells, as well as in their metabolic profile (Guinney *et al*, 2015; Dienstmann *et al*, 2017; Rodriguez‐Salas *et al*, 2017; Soldevilla *et al*, 2019).

Spatial and temporal modulation of Wnt signaling is important for stem cell maintenance and tissue regeneration in the human colon. As such, aberrant activation of the canonical Wnt pathway is the initiating event of the classical model of Wnt‐dependent colorectal tumorigenesis (reviewed in Polakis, 2012). Oncogenic Wnt hyperactivation is highly context‐dependent and can be initiated by different mechanisms including mutations in Wnt pathway components, epigenetic modifications, or alteration of Wnt ligand secretion (reviewed in Flanagan *et al*, 2019). According to the “just‐right” hypothesis, these oncogenic events select for an optimal dosage level of Wnt signaling that is sufficient for cell transformation, but not excessive or cytotoxic (Albuquerque *et al*, 2002). In human colorectal cancer, the most frequent genetic alteration leading to Wnt hyperactivation is an allelic loss or a loss‐of‐function mutation in the Wnt‐regulator *APC* (> 50 percent of tumors; Cancer Genome Atlas Network, 2012; Zhan *et al*, 2017). The truncations in the APC protein occur most frequently before or within the first few repeats that mediate binding to CTNNB1, which is the key transcriptional regulator of Wnt signaling (Polakis, 1995). The truncated APC proteins have a reduced capacity to stabilize the CTNNB1 destruction complex which results in higher CTNNB1‐mediated transcription. The level of Wnt activation and subsequent transcriptional activation is dosage dependent on the number of CTNNB1 binding repeats that remain in the truncated APC protein (Voloshanenko *et al*, 2013). The length distribution of truncated APC proteins in CRC, where the total number of CTNNB1 binding repeats remaining in both *APC* alleles trends toward an optimum, is one of the strongest indications for the “just‐right” hypothesis (Albuquerque *et al*, 2002).

One of the hallmarks of cancer is the reprogramming of energy metabolism (Hanahan & Weinberg, 2011). Tumors are frequently subject to the Warburg effect (reviewed in Hsu & Sabatini, 2008), which is the relative shift from mitochondrial respiration and oxidative phosphorylation (OXPHOS) toward glycolysis as a source of energy. Increasing evidence indicates an intricate interplay between Wnt signaling, metabolism, and mitochondria in colorectal cancer development. On the one hand, mitochondrial pyruvate metabolism plays an essential role in controlling intestinal stem cell proliferation (Schell *et al*, 2017), and several studies report a direct role of Wnt signaling in biogenesis, maintenance, and physiology of mitochondria (Yoon *et al*, 2010; Brown *et al*, 2017; Bernkopf *et al*, 2018). On the contrary, Wnt signaling maintains increased glucose metabolism (Lee *et al*, 2012) and directly regulates the transcription of important mediators of glycolysis and pyruvate metabolism such as *PDK1* and *MCT1* (Pate *et al*, 2014). As such, Wnt signaling is needed to maintain a beneficial metabolic equilibrium in tumors with Wnt hyperactivity (Pate *et al*, 2014; Yang *et al*, 2014). A recent study in mice showed that the loss of *APC* and the resulting Wnt hyperactivation leads to an increased glucose uptake not only in tumors, but also in intestinal tissue (Najumudeen *et al*, 2021).

Different levels of Wnt hyperactivation and metabolic dysregulation have been reported for the CMS of CRC (Guinney *et al*, 2015; Fessler & Medema, 2016). However, the role of these different levels of Wnt signaling activity during tumorigenesis and how they influence other CMS characteristics such as metabolism or immune infiltration remains unclear. In this study, we explored the role of Wnt signaling in the development of tumors of the different CMS classes. We performed an integrative multi‐omic analysis of transcriptomic, proteomic, and large‐scale genetic perturbation data from tumor tissue and colorectal cancer cell lines to investigate functional heterogeneity of colorectal cancer. We showed that tumor development that involves strong Wnt hyperactivation leads to a different metabolic state than tumor development that only involves low Wnt activity. To explore the context‐dependent effects of Wnt signaling, we introduced *APC* loss‐of‐function mutations in *APC* ^WT^ colorectal cancer cells and performed whole‐genome perturbation screens in these genome‐engineered model systems. We show that cells with engineered Wnt hyperactivation have a different metabolomic state than cells that naturally acquired Wnt hyperactivation. In summary, our data indicate that the effect of Wnt signaling activation is dependent on the baseline metabolic state of a cell and thereby exemplifies context‐dependency in genetic networks.

## Results

### Distinct molecular features of Wnt‐low and Wnt‐high CRC

**Figure 1:**
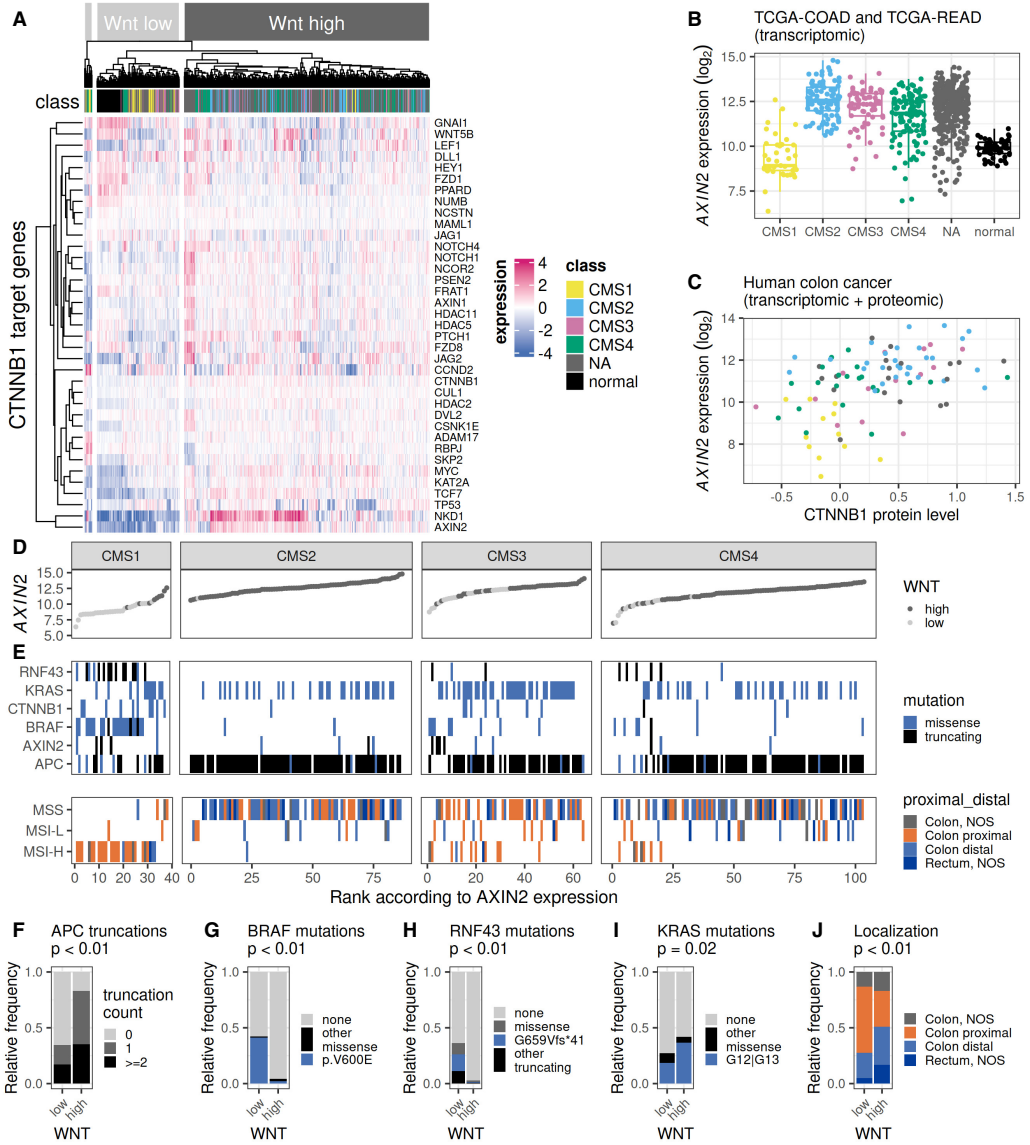
Distinct molecular and clinical features of Wnt‐low and Wnt‐high colorectal cancers AHeatmap of the expression of CTNNB1 target genes (MsigDb pathway HALLMARK_WNT_BETA_CATENIN_SIGNALING) in the tumor and normal tissue samples from TCGA‐COAD and TCGA‐READ cohorts (Guinney *et al*, 2015). Clustering into Wnt‐low (light gray) and Wnt‐high (dark gray) groups was performed using k‐means clustering. The bar entitled “class” indicates a normal tissue origin (black) or the result from CMS classification (tumor samples only, NA: no CMS could be assigned). TCGA tumor samples were classified into different consensus molecular subtypes (CMS) using RNA sequencing data and the R package CMSclassifier (Guinney *et al*, 2015).B
*AXIN2* RNA expression in TCGA samples classified into the different CMS as well as normal colon tissue. Represented data correspond to TCGA‐COAD and TCGA‐READ cohorts (Guinney *et al*, 2015). Individual data points and box plots are displayed. Box plots consist of the median (central line), the 25^th^ and 75^th^ percentiles (box) and the highest/lowest value within 1.5 * interquartile range of the box (whiskers). Each data point corresponds to a different tumor or normal (tumor‐adjacent) tissue sample.CCorrelation between *AXIN2* RNA expression and CTNNB1 protein level in samples from the 2019 CPTAC‐COAD proteomics cohort (Vasaikar *et al*, 2019). CMS classification is represented in color and is assigned according to the original publication. Protein level corresponds to fold change of tumor protein abundance versus adjacent normal tissue protein abundance.D, ETumor samples in the different CMS were ranked according to their *AXIN2* RNA expression. Panel D depicts the *AXIN2* RNA expression as a function of the sample rank. (E) Tile plot of TCGA tumor samples classified into the four CMS. Upper panel: presence of protein missense (blue) or truncating (black) mutation in different tumor driver and suppressor genes: *RNF43*, *KRAS*, *CTNNB1*, *BRAF*, *AXIN2*, and *APC*. Lower panel: Anatomical origin (proximal or distal colon, color coded) of tumors classified according to their DNA mismatch repair phenotype into microsatellite instability high (MSI‐H), microsatellite instability low (MSI‐L) or microsatellite stable (MSS). Proximal colon: cecum, ascending colon, hepatic and splenic flexure, transverse colon. Distal colon: descending and sigmoid colon. NOS: no more precise origin specified.F–IFrequency of cancer driver mutations in Wnt‐low (*n* = 106 samples) and Wnt‐high tumors (*n* = 432 samples) classified according to (A). *P*‐values from Fisher's exact test for independence are indicated. (F) Relative frequency of tumors with 0, 1 or more truncating APC mutations annotated in each of the tumor groups. Allelic loss of *APC* was not considered here. (G) Relative frequency of BRAF V600E (blue) or other BRAF missense mutations (black). (H) Relative frequency of missense mutations (gray), G659Vfs*41 (blue) and other truncating (black) mutations in the RNF43 protein. (I) Relative frequency of missense mutations at residues G12 or G13 (blue) or other residues (black) of the KRAS protein.JFrequency of tumor localization in Wnt‐low (*n* = 106 samples) and Wnt‐high tumors (*n* = 432 samples) classified according to (A). Colors as in (E), lower panel. *P*‐values from Fisher's exact test for independence are indicated.

To gain a detailed understanding of the role of different levels of classical Wnt signaling in CRC, we first explored the transcriptomic and proteomic Wnt signatures in tumor and normal tissue samples from the TCGA‐COAD, TCGA‐READ, and CPTAC‐COAD cohorts (Guinney *et al*, 2015; Vasaikar *et al*, 2019). The expression of classical CTNNB1‐dependent transcriptional Wnt targets such as *AXIN2* and *NKD1* was elevated in a large fraction of tumor samples compared with normal tissue (Fig 1A–C). As expected, elevated *AXIN2* expression levels correlated with elevated CTNNB1 protein levels, indicating a stabilization of CTNNB1 in agreement with hyperactivation of Wnt‐dependent transcription (Fig 1C). In accordance with previous studies, the CMS1 subtype was enriched for tumors that lack signs of Wnt hyperactivation (Fig 1A and B). However, the CMS classification was not sufficient to predict Wnt activation, as average *AXIN2* expression levels comparable with normal colon tissue were also found in a subset of CMS4 and CMS3 tumors (Fig 1A and B and Appendix Fig S1). We therefore classified the tumors into Wnt‐high or Wnt‐low tumors based on their expression of classical CTNNB1 target genes (Fig 1A and D). This classification also included tumor samples for which CMS assignment was ambiguous (annotated as “NA” in Fig 1A–C).

Different frequencies of well‐described CRC‐driver mutation could be observed in the Wnt‐high and Wnt‐low tumors, indicating a different sequence of events leading to tumor development in the two groups. Around 80% of Wnt‐high tumors carried one or more truncating *APC* mutations, whereas this was only the case for around 30% of Wnt‐low tumors (Fig 1E and F). Wnt‐low tumors had a high prevalence (40%) of the characteristic BRAF V600E mutation, which was almost not observed in Wnt‐high tumors (Fig 1E and G). Similarly, mutations in the Wnt regulator RNF43 and in particular G659Vfs*41 mutations were enriched in Wnt‐low tumors (Fig 1E and H). The prevalence of KRAS missense mutations, and specifically mutations in residues G12 and G13, was only slightly reduced in Wnt‐low tumors compared with Wnt‐high tumors (25 versus 40%, Fig 1E and I). Simultaneous existence of BRAF missense mutation and an APC truncation was rarely observed, which further supported the hypothesis that the most important oncogenic driver events differed between Wnt‐low and Wnt‐high tumor groups (Fig 1E). In summary, the high expression level of Wnt‐dependent targets in Wnt‐high tumors seems to be linked to alterations in *APC*, whereas the mutations in *RNF43* observed in Wnt‐low tumors do not lead to strong hyperactivation of Wnt‐dependent transcription.

Wnt‐high and Wnt‐low tumors also exhibited differences in genetic stability and tumor localization. Wnt‐low tumors accumulated in the proximal part of the colon (Fig 1E and J) and frequently harbored a deficiency in DNA mismatch repair (MSI, microsatellite instability, Fig 1E). Wnt‐high tumors in contrast rarely showed signs of DNA mismatch repair deficiency and developed in both proximal and distal parts of the colon (Fig 1E and J). In summary, our classification of tumors into Wnt‐high and Wnt‐low allowed us to delineate two groups of tumors with distinct mutational and molecular patterns. Differences in characteristic features such as tumor localization and DNA mismatch repair deficiency adequately reflect the idea of independent routes of tumorigenesis, also known as the serrated pathway (~Wnt‐low) and the canonical adenoma‐carcinoma‐pathway (~Wnt‐high; Nguyen *et al*, 2020).

### Classification of CRC cell lines into Wnt‐low and Wnt‐high groups

**Figure 2:**
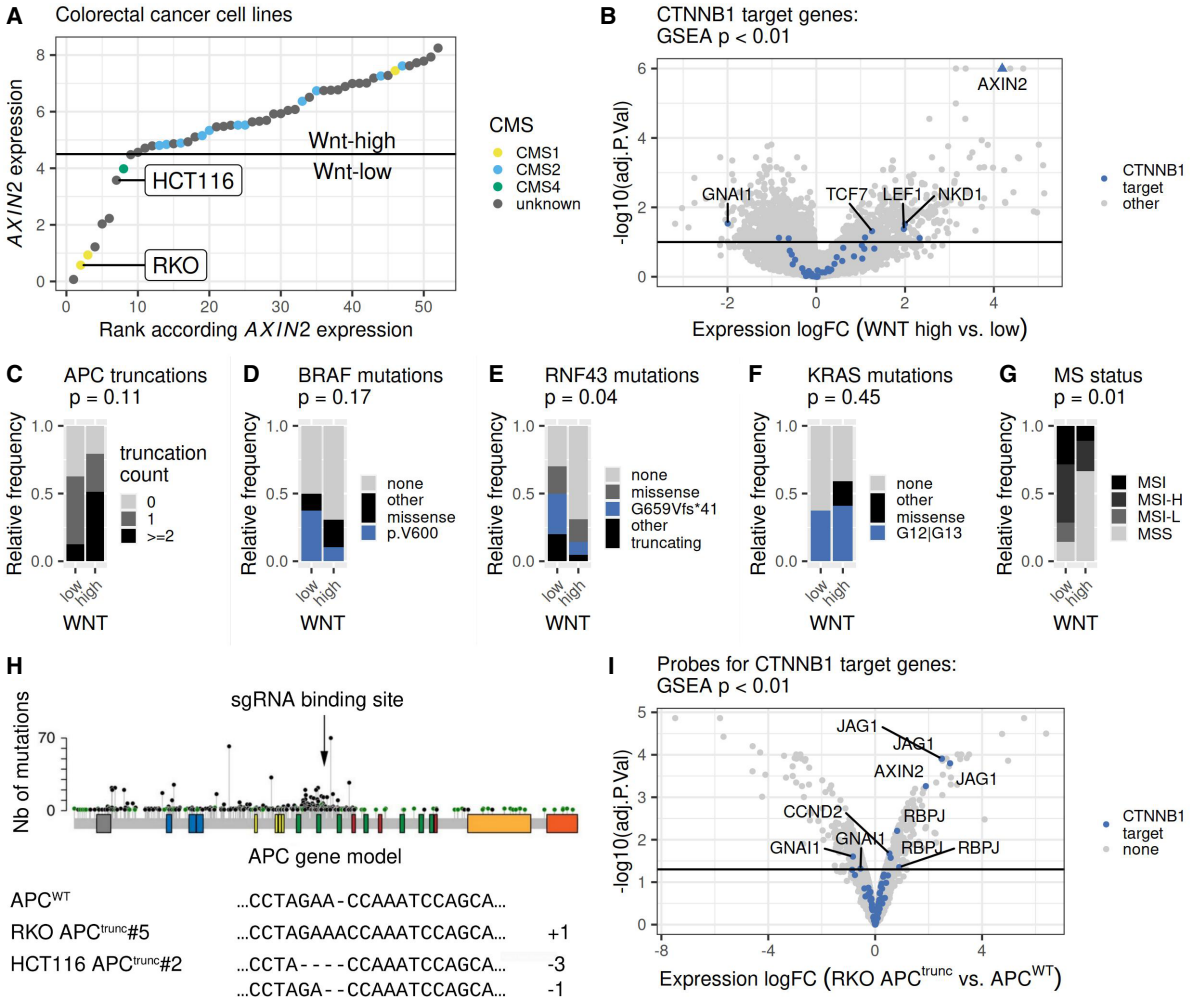
CRC cell lines to model context‐dependent Wnt signaling in Wnt‐high and Wnt‐low tumors AColorectal cancer cell lines were ranked according to *AXIN2* RNA expression and classified into Wnt‐high and Wnt‐low groups (Ghandi *et al*, 2019). Annotation of CMS for cell lines according to Zhan *et al* (2021). The horizontal line indicates the threshold for classification into Wnt‐low and Wnt‐high groups.BVolcano plot summarizing the results from differential gene expression analysis comparing Wnt‐low (*n* = 8 cell lines) versus Wnt‐high (*n* = 39 cell lines) CRC cell lines. Gene expression data from RNA sequencing (Ghandi *et al*, 2019). Genes with higher expression in Wnt‐high cell lines have positive fold changes. The horizontal line indicates an adjusted *P*‐value threshold of 0.1, which was calculated as described in the Materials and Methods section. CTNNB1 target genes (MsigDb pathway HALLMARK_WNT_BETA_CATENIN_SIGNALING) are highlighted in blue. Gene set enrichment analysis for CTNNB1 target genes indicated a positive enrichment score with an adjusted *P*‐value < 0.01.C–FFrequency of cancer driver mutations in Wnt‐low (*n* = 8) and Wnt‐high (*n* = 39) CRC cell lines classified according to (A). The different cell lines represent independent biological replicates. *P*‐values from Fisher's exact test for independence are indicated. (C) Relative frequency of cell lines with 0, 1 or more truncating APC mutations in each of the groups. (D) Relative frequency of BRAF V600E (blue) or other BRAF missense mutations (black). (E) Relative frequency of missense mutations (gray), G659Vfs*41 (blue) and other truncating (black) mutations in the RNF43 protein. (F) Relative frequency of missense mutations at residues G12 or G13 (blue) or other residues (black) of the KRAS protein.GRelative frequency of cell lines with a given microsatellite stability status in Wnt‐low (*n* = 8) and Wnt‐high (*n* = 39) CRC cell lines classified according to (A). The different cell lines represent independent biological replicates. *P*‐value from Fisher's exact test for independence is indicated. MSI, microsatellite instable; MSI‐H, microsatellite instability high; MSI‐L, microsatellite instability low; MSS, microsatellite stable. Assignment of microsatellite stability status according to Zhan *et al* (2021).HIntroduction of APC truncations in RKO (Zhan *et al*, 2019) and HCT116 cells using CRISPR/Cas9. Correct gene editing and successful APC truncation in single‐cell clones RKO‐APC^trunc^#5 and HCT116‐APC^trunc^#2 was confirmed by targeted amplicon sequencing of the edited gene locus.IVolcano plot summarizing the results from differential gene expression analysis comparing RKO‐APC^trunc^#5 versus RKO cell lines. Gene expression was assessed using microarrays in two biological replicates per cell line. The horizontal line indicates an adjusted *P*‐value threshold of 0.05, which was calculated as described in the Materials and Methods section. Genes with higher expression in RKO‐APC^trunc^#5 cells have positive fold changes. Colors as in (B). Gene set enrichment analysis for CTNNB1 target genes indicated a positive enrichment score with a *P*‐value < 0.01 calculated.

We next confirmed that a similar classification into Wnt‐high and Wnt‐low entities can be applied to CRC cell lines based on *AXIN2* expression levels (Fig 2A). CMS classification is more difficult for cell lines than for tumor tissues (Eide *et al*, 2017; Linnekamp *et al*, 2018; Zhan *et al*, 2021), presumably due to lack of immune and stromal infiltration in cell lines, which contribute to a certain degree to the transcriptional signature of CMS in tumors. Nevertheless, cell lines with CMS1 annotation were mostly found in the Wnt‐low group, and cell lines with CMS2 annotation were exclusively part of the Wnt‐high group (Fig 2A). As expected, classical CTNNB1‐dependent Wnt target genes had a tendency to be higher expressed in the Wnt‐high cell lines compared with Wnt‐low cell lines (Fig 2B). The genes most differentially expressed in the analysis of cell lines were the same genes as the ones with strong expression differences between Wnt‐high and Wnt‐low tumor samples, namely *GNAI1*, *NKD1*, and *TCF7* (Figs 1A and 2B).

The patterns of cancer driver mutations in Wnt‐low and Wnt‐high colorectal cancer cell lines were partially comparable with the ones observed in the tumor tissue (Fig 2C–F). RNF43 mutations were more frequently observed in Wnt‐low cell lines (Fig 2E). Wnt‐low colorectal cancer cell lines were more likely to manifest a MSI‐phenotype than Wnt‐high cell lines, which was also in accordance with our findings for the tumor tissue (Figs 2G and 1E). The differences in APC truncations, BRAF and KRAS missense mutations were not significant (Fig 2C, D, and F).

### Wnt activation in Wnt‐low CRC cell lines using CRISPR‐Cas9‐mediated APC truncation

We next engineered an additional cell line system to explore the context‐dependent effects of Wnt hyperactivation in CRC (Appendix Fig S2A). We selected two cell lines with low endogenous Wnt activation and without endogenous *APC* mutations (HCT116 and RKO, APC^WT^, Fig 2A). To study the effect of Wnt hyperactivation in these cells, we genetically engineered a truncating mutation of the *APC* gene in both HCT116 and RKO cell lines. For this, we used CRISPR/Cas9 in combination with an sgRNA targeting a specific region of the *APC* gene that frequently harbors protein‐truncating somatic mutations in colorectal cancers (Fig 2H and Appendix Fig S2A). The engineered truncation of APC in the resulting isogenic cell lines HCT116‐APC^trunc^ and RKO‐APC^trunc^ was verified by amplicon sequencing (Fig 2H) and by Western blot analysis (Appendix Fig S2B). The corresponding results regarding RKO‐APC^trunc^ cells were previously described by Zhan *et al* (2019), whereas results for both cell lines are summarized in Appendix Fig S2.

As expected, HCT116‐APC^trunc^ and RKO‐APC^trunc^ showed a higher level of *TCF4*/Wnt‐reporter activity than HCT116 and RKO cells (Appendix Fig S2C and Zhan *et al*, 2019). In the same lines, the expression of classical CTNNB1‐dependent Wnt target genes was elevated in RKO‐APC^trunc^ compared with RKO cells (Fig 2I). However, the Wnt target genes most upregulated upon engineered Wnt hyperactivation in RKO cells (*JAG1* and *RBPJ*) were not the same as the Wnt target genes with higher expression in cell lines and tumors with endogenous Wnt hyperactivation (Figs 1A, and 2B and I). These results highlight the context‐dependent transcriptional effects of Wnt hyperactivation. The overall molecular mechanisms of Wnt‐dependent transcription activation are comparable between different cellular contexts, as indicated by the *TCF4*/Wnt‐reporter assay (Zhan *et al*, 2019; Appendix Fig S2C). The actual transcriptional changes, however, differ due to the context‐dependent genetic interaction networks, where for example, the target genes of a given transcription factor depend on the chromatin status in a specific context. Indeed, our results show that the list of genes upregulated upon endogenous Wnt hyperactivation during tumorigenesis was partially distinct from the list of genes upregulated upon artificial Wnt hyperactivation in cell lines without endogenous signaling (Figs 1A, and 2B and I).

### Differential genetic dependencies in natural and engineered APC mutant cells

**Figure 3:**
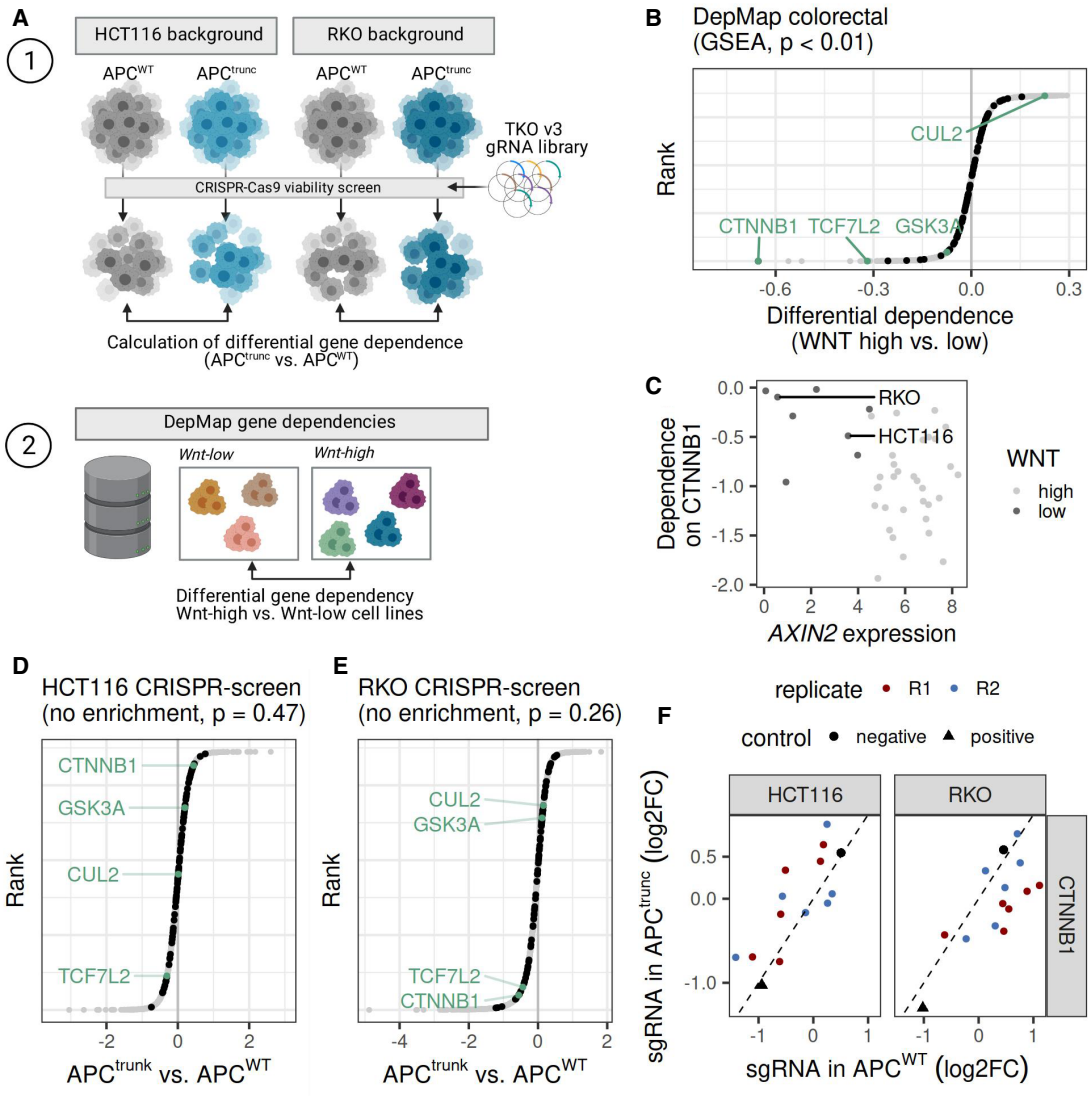
Hyperactivation of Wnt signaling in Wnt‐low cells does not recapitulate Wnt dependency of Wnt‐high cells A(1) Schematic representation of whole‐genome CRISPR screen to compare gene essentiality in APC^trunc^ and APC^WT^ RKO and HCT116 cell lines. Differential gene essentiality was assessed by comparing the gRNA abundances at T1 in APC^trunc^ and APC^WT^ cell pools in both RKO and HCT116 backgrounds. The screen was conducted in two replicates per cell line. Doubling times for RKO‐APC^WT^: 23.8 h; RKO‐APC^trunc^#5: 23.9 h; HCT116‐APC^WT^: 21.0 h; HCT116‐APC^trunc^#2: 25.7 h. (2) DepMap data was used to assess the differential gene essentially in Wnt‐high (*n* = 24 cell lines) versus Wnt‐low (*n* = 5 cell lines) CRC cell lines (classification as in Fig 2A). Created with Biorender.com.BDifferential gene essentiality in Wnt‐high versus Wnt‐low CRC cell lines displayed as logarithmic fold change (logFC). Negative logFC indicate genes that are more essential in Wnt‐high compared to Wnt‐low cell lines. Genes involved in Wnt signaling are highlighted in black (MSigDB curated gene set WNT_SIGNALING; Liberzon *et al*, 2011). Gene set enrichment analysis using the permutation based statistical test implemented in the fgsea R package (preprint: Korotkevich *et al*, 2021) and Benjamini–Hochberg correction for multiple testing indicated a positive enrichment score for absolute logarithmic fold changes (*P*‐value < 0.01) for this pathway. Selected genes of interest belonging to this pathway are highlighted and labeled in green.CDependency on *CTNNB1* as a function of *AXIN2* expression in Wnt‐high (light gray) and Wnt‐low (dark gray) CRC cell lines. The Wilcoxon‐rank sum test indicated that the dependence on *CTNNB1* is different in Wnt high compared to Wnt low cells with a *P*‐value < 0.01.D, EDifferential gene essentiality in APC^trunc^ versus APC^WT^ HCT116 (D) and RKO (E) cell lines displayed as logarithmic fold change (logFC). Negative logFC indicates genes that are more essential in APC^trunc^ compared with APC^WT^ cells. Gene set enrichment analysis using the permutation based statistical test implemented in the fgsea R package (preprint: Korotkevich *et al*, 2021) and Benjamini–Hochberg correction for multiple testing indicated no significant enrichment for absolute logarithmic fold changes of genes involved in Wnt signaling. Gene set definition, color and labeling as in (B).FViability effects of single gRNAs targeting *CTNNB1*. Logarithmic fold changes of gRNA abundance in the screening endpoint compared to the plasmid library are displayed for APC^trunc^ (*y*‐axis) and APC^WT^ (*x*‐axis) for HCT116 (left panel) and RKO (right panel) cells. Red and blue circles indicate results for replicates 1 and 2 of the CRISPR screen. Mean fold changes of negative control gRNAs (targeting luciferase) are displayed as black circles. Mean fold changes of positive control gRNAs (chromosome 10 promiscuous) are displayed as black triangles.

We next used functional genetic and gene dependency data derived from whole‐genome CRISPR/Cas9 viability screens to further explore the context‐dependent effects of Wnt hyperactivation on a functional level. This analysis consisted of two different approaches (Fig 3A). In the first approach, we performed four different whole‐genome CRISPR viability screens in the four isogenic cell lines HCT116, RKO, HCT116‐APC^trunc^, and RKO‐APC^trunc^ (Fig 3A and Appendix Fig S3A–C). We used the CRISPR library for screening, which contains around 90,000 gRNAs targeting more than 17,000 genes. We calculated the differential viability effect of gene knockout in APC^trunc^ versus APC^WT^ cell lines in both RKO and HCT116 backgrounds (Appendix Fig S3D). We used a statistical model that accounts for skewed fold change distributions for the comparison of gRNA abundances, so that the results would not be affected by differences in editing efficiencies or cellular growth rates (Imkeller *et al*, 2020). This first approach allowed us to explore the genetic rewiring in Wnt‐low cancer cell lines upon Wnt hyperactivation. In the second approach, we reanalyzed gene dependency data from the DepMap CRISPR/Cas9 screening project to assess the functional differences between colorectal cancer cells that did or did not undergo natural Wnt hyperactivation during tumorigenesis. In this approach, we also calculated the differential viability effect of gene knockout in Wnt‐high compared with Wnt‐low colorectal cancer cell lines (groups as defined in Fig 2A). The combination of both approaches allowed us to compare the functional effects of endogenous Wnt hyperactivation to those of engineered Wnt hyperactivation in Wnt‐low cell line models.

When analyzing the differential gene dependencies within the DepMap project, we found that Wnt‐high colorectal cancer cell lines were more dependent on *CTNNB1* and other members of the Wnt signaling pathway than Wnt‐low colorectal cancer cell lines (Fig 3B and C). In CRISPR screens in our engineered APC^trunc^ system, however, the dependence on components of the Wnt signaling pathway was comparable in APC^trunc^ and APC^WT^ cells in both RKO and HCT116 backgrounds (Fig 3D–F), indicating that introducing an APC truncation in Wnt‐low colorectal cancer cell lines, and thereby hyperactivating Wnt signaling, did not lead to a new dependency on Wnt signaling in the resulting APC^trunc^ cell lines. Conversely, and in contrast to the effect of naturally occurring Wnt hyperactivation during tumorigenesis, APC truncation did not lead to an increase in Wnt‐dependency in HCT116‐APC^trunc^ and RKO‐APC^trunc^ cell lines compared with HCT116 and RKO cell lines. Of note, the overall vulnerability toward knockout of *CTNNB1* was higher in the two HCT116 cell line variants compared with the two RKO cell line variants, probably due to the fact that HCT116 have a low endogenous Wnt activation resulting from a mutation in *CTNNB1* (Fig 3F).

### Engineered APC^trunc^ cells acquire vulnerability toward metabolic and mitochondrial perturbation

In a next step, we further explored the differences in genetic dependencies in the three groups of cell lines, namely endogenously Wnt‐high, endogenously Wnt‐low, and endogenously Wnt‐low with engineered APC truncation.

**Figure 4:**
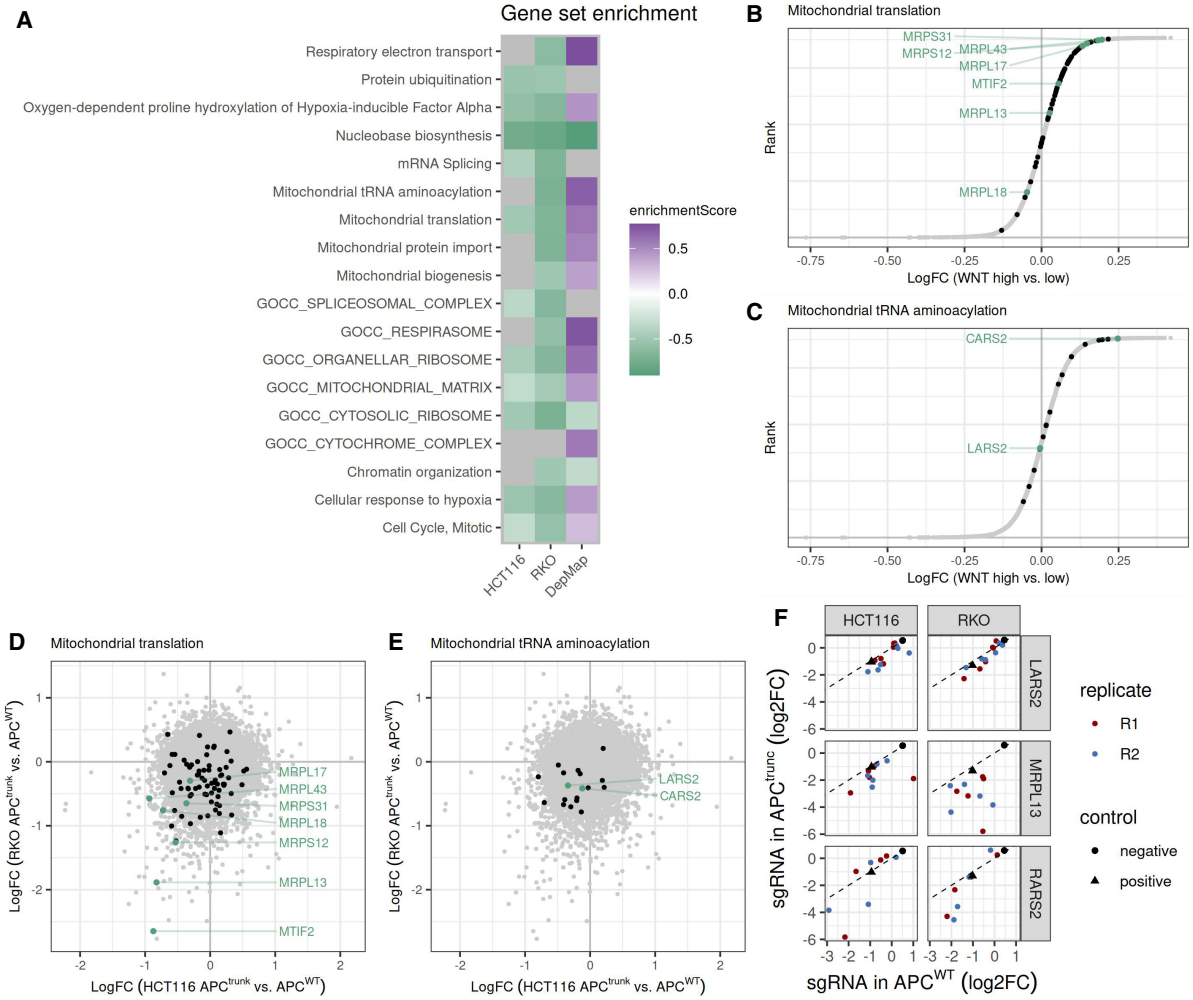
Different metabolic dependencies of Wnt‐high and Wnt‐low CRC cell lines with or without Wnt hyperactivation ASelection of gene set enrichment analysis results for CRISPR screening results in our engineered cell lines as well as in the DepMap datasets. Gene sets for which the enrichment analysis indicated adjusted *P*‐values > 0.05 are depicted in gray. Negative enrichment scores in green indicate a higher essentiality of the respective gene sets in APC^trunc^ compared with APC^WT^ cell lines (HCT116 and RKO) or Wnt‐high compared to Wnt‐low (DepMap).B, CDifferential gene essentiality in Wnt‐high versus Wnt‐low cell lines (DepMap project) displayed as logarithmic fold change (logFC). Negative logFC indicates genes that are more essential in Wnt‐high cell lines compared with Wnt‐low cell lines. Genes involved in mitochondrial translation (E) and mitochondrial tRNA aminoacylation (F) are highlighted in black. Gene set enrichment analysis indicated a positive enrichment score for both pathways. Genes involved in mitochondrial translation (B, Reactome R‐HSA‐5368287) and mitochondrial tRNA aminoacylation (C, Reactome R‐HSA‐379726) are highlighted in black. Selected genes of interest belonging to this pathway are highlighted and labeled in green.D, ECorrelation of differential gene essentialities in APC^trunc^ versus APC^WT^ HCT116 (*x*‐axis) and RKO (*y*‐axis) cell lines displayed as logarithmic fold change (logFC). Negative logFC indicates genes that are more essential in APC^trunc^ compared to APC^WT^ cells. Gene set definition, color and labeling as in (B, C).FViability effects of single gRNAs targeting *LARS2*, *MRPL13* and *MTIF2*. Logarithmic fold changes of gRNA abundance in the screening endpoint compared to the plasmid library are displayed for APC^trunc^ (*y*‐axis) and APC^WT^ (*x*‐axis) for HCT116 (left panel) and RKO (right panel) cells. Red and blue circles indicate results for replicates 1 and 2 of the CRISPR screen. Mean fold changes of negative control gRNAs (targeting luciferase) are displayed as black circles. Mean fold changes of positive control gRNAs (chromosome 10 promiscuous) are displayed as black triangles.

We first performed gene set enrichment analysis on the differential fitness effects in the DepMap project to compare endogenously Wnt‐high and endogenously Wnt‐low cell lines. Cell lines with low endogenous Wnt signaling were more dependent on metabolic pathway genes and genes involved in mitochondrial function than Wnt‐high cell lines (Fig 4A–C and Appendix Fig S4). Among the genes with highest differential viability effect, we found components of the mitochondrial transcription machinery such as *MTIF2* (mitochondrial translation initiation factor 2), mitochondrial ribosomal proteins such as *MRPL13*, *MRPS12*, and mitochondrial tRNA synthetases such as *CARS2* (Fig 4B and C). These components are encoded in the nucleus, translated in the cytoplasm, and then imported into the mitochondrion, where they participate in the translation of mitochondrially encoded proteins essential for mitochondrial function and oxidative phosphorylation (reviewed in Kummer & Ban, 2021).

We then used the results from our CRISPR viability screens and performed gene set enrichment analysis on the differential gene dependencies in APC^trunc^ compared with APC^WT^ cells in both HCT116 and RKO backgrounds. HCT116‐APC^trunc^ and RKO‐APC^trunc^ cell lines were more dependent on metabolic pathway genes and genes involved in mitochondrial function than HCT116 and RKO cell lines (Fig 4A and Appendix Fig S4). When focusing on the mitochondrial translation and tRNA aminoacylation pathways and comparing the genes with highest differential dependency, we found that they were the same genes in both RKO and HCT116 backgrounds, namely *MTIF2*, *MRPL13*, *CARS2*, and another mitochondrial tRNA synthetase *LARS2* (Fig 4D–F).

Taken together, these results indicate that engineered Wnt hyperactivation in Wnt‐low HCT116 and RKO cell lines did not recapitulate the metabolic phenotype of cell lines that underwent natural Wnt hyperactivation. In fact, the three groups of cell lines that we analyzed, namely endogenously Wnt‐high, endogenously Wnt‐low, and endogenously Wnt‐low with engineered APC truncation, represented three different states of metabolic and mitochondrial dependence. Wnt‐high cell lines were least affected by mitochondrial perturbation. Wnt‐low cell lines in turn had an intermediate level of mitochondrial dependence that became even more accentuated when Wnt signaling was artificially hyperactivated after the introduction of an APC truncation (Wnt‐low cells with engineered APC truncation).

It is important to note that the knockout of genes involved in mitochondrial function induced a measurable fitness defect in all analyzed cell lines (Fig 4D–F). However, we were able to reproducibly detect differential dependency in all analyses, as described above, which indicates different cell lines can be more or less dependent on essential pathways such as mitochondrial function. The increased vulnerability toward mitochondrial perturbation was not linked to increased cellular growth rate, as in both RKO and HCT116 backgrounds, the APC^trunc^ cells grew either at similar speed or at slower than the APC^WT^ cells (Fig 3A, figure legend).

### Synthetic mitochondrial vulnerability in engineered APC^trunc^ cells

Among the genes involved in mitochondrial function, some had an intermediate viability effect in all cell lines (Fig 4D and E), which made them suitable for use in functional assays, where a minimum of cell viability is necessary. We selected *LARS2* as an exemplary candidate gene to further explore the mechanisms of vulnerability to mitochondrial perturbation.

**Figure 5:**
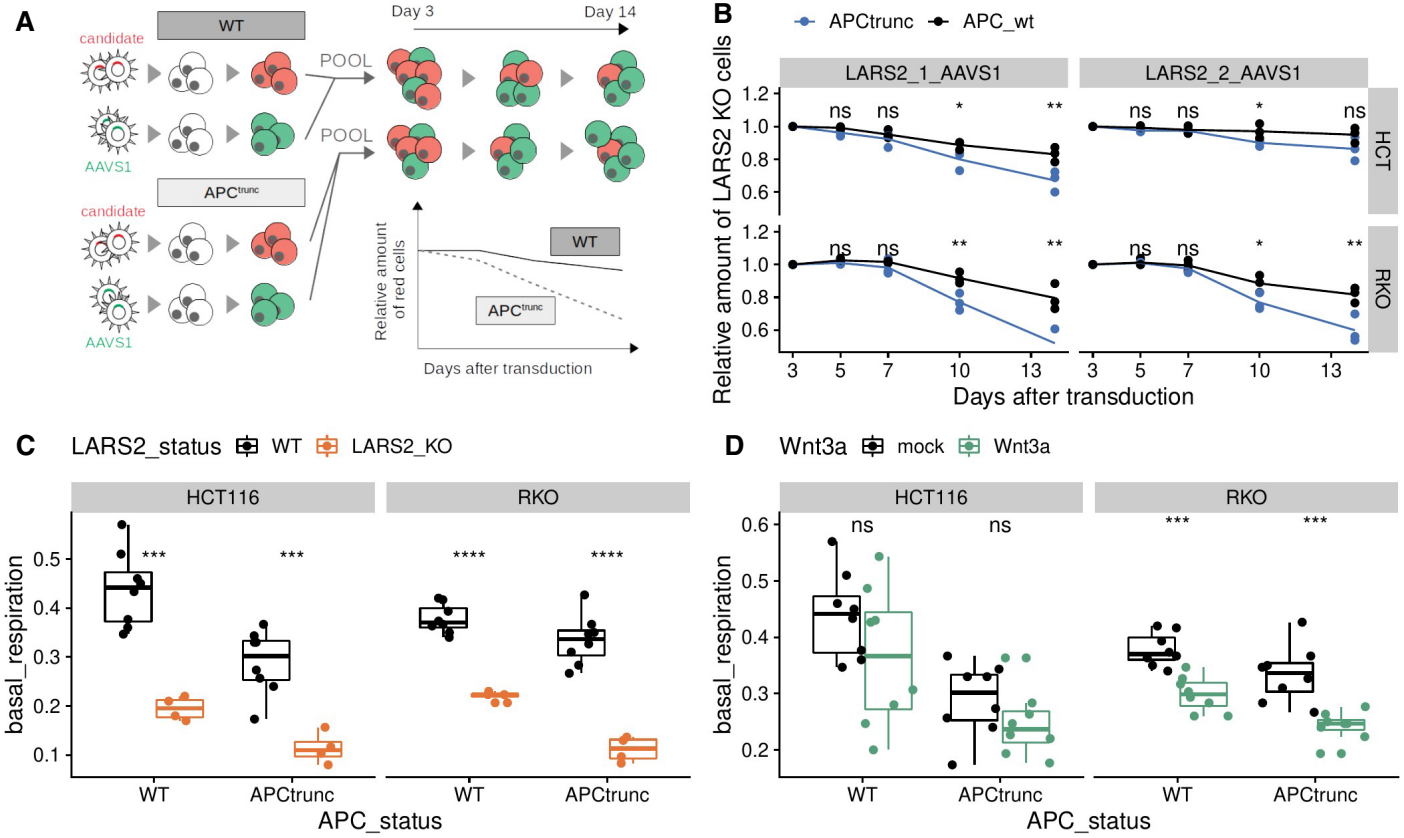
Perturbation of mitochondrial tRNA synthesis affects APC^trunc^ cell lines stronger than APC^WT^ cells. The mitochondrial insufficiency phenotype is Wnt‐dependent AExperimental setup of competitive growth assay.BRelative amount of *LARS2* KO cells compared to *AAVS1* KO cells, normalized to the proportions at day 3 after pooling. Pools of APC^trunc^ cells are shown in blue, pools of APC^WT^ cells are shown in black. The complete assay was repeated three times. The dots represent results from the individual replicates, whereas the line connects the mean values over all three replicates per time point.C, DBasal respiration derived from Seahorse oxygen consumption rate measurement. *LARS2* KO samples are highlighted in orange (C). Wnt3a treated samples are highlighted in green (D). Box plots consist of the median (central line), the 25^th^ and 75^th^ percentiles (box) and the highest/lowest value within 1.5 * interquartile range of the box (whiskers). Every dot represents one technical replicate, which were performed in at least two different experimental batches. Data information: Statistical tests in all: paired *t*‐test corrected for multiple testing, ****: *P* < 0.001, ***: *P* < 0.01, **: *P* < 0.05, *: *P* < 0.1.

We first used a fluorescence and flow cytometry‐based competitive cell growth assay to compare the growth rates of cells treated with *LARS2* targeting guide RNAs to those treated with guide RNAs targeting the safe harbor locus *AAVS1* locus (negative control). Using sgRNA expression vectors with fluorescence markers, we labeled *LARS2* knockout cells in red and control cells in green. For all four HCT116 and RKO cell line variants, the green and red cells were pooled at equal proportions and the proportion of red (*LARS2* knockout cells) monitored over the course of 2 weeks (Fig 5A). We confirmed that APC^trunc^ cells were more vulnerable to the knockout of *LARS2* than APC^WT^ cells in both RKO and HCT116 backgrounds (Fig 5B). This result was reproducible using two different *LARS2* targeting gRNAs (Fig 5B).

We next quantified the basal respiration in the cell lines using oxygen consumption measurements. Knockout of the mitochondrial tRNA synthetase *LARS2* reduced the basal respiration in both APC^WT^ and APC^trunc^ cells (Fig 5C), which was likely the cause of decreased growth rate in all cell lines. Although the reduction in growth rate upon *LARS2* perturbation was significantly stronger in APC^trunc^ than in APC^WT^ cell lines (Fig 5B), the basal respiration was strongly reduced in both cell lines (Fig 5C). The APC^trunc^ cell lines thus seemed to have a reduced capacity to compensate for the loss of mitochondrial function.

It has previously been reported that Wnt signaling is able to induce metabolic changes in line with the Warburg effect in colorectal cancer cells (Pate *et al*, 2014). Indeed, we observed that APC^trunc^ cells had a slightly lower basal respiration than APC^WT^ cell lines in both RKO and HCT116 backgrounds. To investigate whether this effect was directly dependent on Wnt signaling, we measured basal respiration rates after external stimulation of Wnt signaling. The addition of Wnt3a ligands into the growth medium resulted in significant reduction in basal respiration in both RKO cell lines (Fig 5D). For HCT116 cell lines, there was no significant reduction in basal respiration upon Wnt3a treatment, which could be due to the fact that HCT116 cells already exhibit a low Wnt activation due to an activating mutation in *CTNNB1* (Fig 5D).

### Different metabolic equilibria in Wnt‐low and Wnt‐high tumors

Our results suggest that engineered APC^trunc^ cells suffer from an unfavorable metabolic equilibrium that is brought about by Wnt hyperactivation due to APC truncation. During Wnt‐dependent tumorigenesis, however, Wnt hyperactivation entails a fitness advantage that results in the development of Wnt‐high tumors. To explain this context‐dependency of the effect of Wnt signaling, we used transcriptomics and proteomics data to characterize the metabolic equilibria in the different tumor types. The analysis included transcriptomic and proteomic data from primary tumor tissue (TCGA and CPTAC data) as well as colorectal cancer cell lines (DepMap data). The tumors and cell lines were classified into Wnt‐high and Wnt‐low entities as described in Figs 1 and 2. The differential expression of transcripts and proteins between Wnt‐low and Wnt‐high groups was quantified separately for tumors and cell lines.

**Figure 6:**
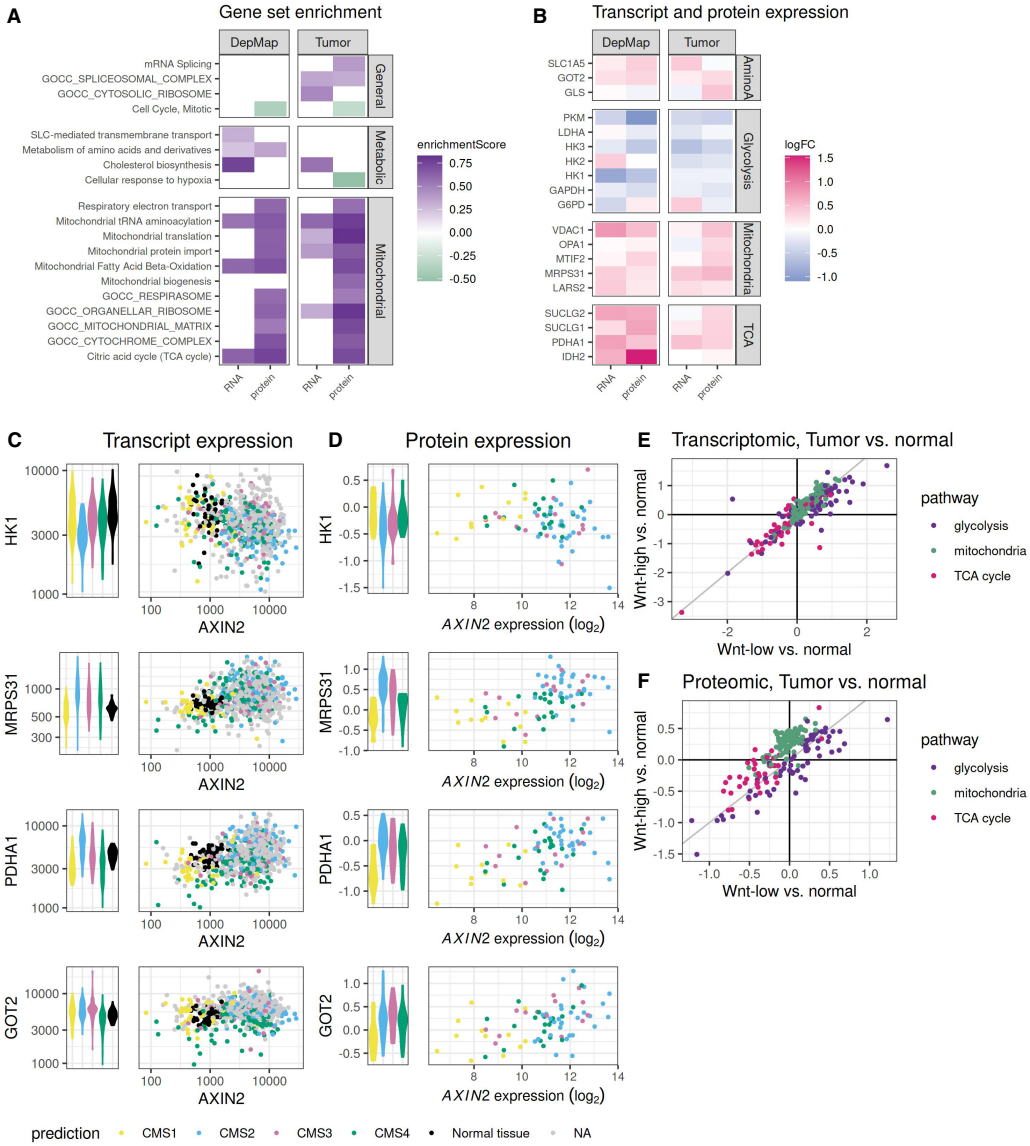
Transcriptomic and proteomic signatures of different metabolic equilibria in Wnt‐low and Wnt‐high tumors ASelection of gene set enrichment analysis results for transcriptomic (*x*‐axis “RNA”) and proteomic (*x*‐axis “protein”) differences between Wnt‐high and Wnt‐low colorectal cancer cell lines (depmap, 39 Wnt‐high and 8 Wnt‐low cell lines for transcriptomics and 16 Wnt‐high and 6 Wnt‐low for proteomic) and tumors (432 Wnt‐high and 108 Wnt‐low for transcriptomics (TCGA), 31 Wnt‐high and 75 Wnt‐low samples for proteomic (CPTAC‐COAD)). Positive enrichment scores in green indicate a higher expression of the respective gene sets in Wnt‐high compared to Wnt‐low entities. Gene set annotation according to Reactome pathways and GO cellular component, an adjusted *P*‐value threshold of 0.05 was applied.BDifferential transcript (*x*‐axis “RNA”) and protein (*x*‐axis “protein”) abundance in colorectal cancer cell lines (depmap) and tumor tissues (TCGA and CPTAC‐COAD). Selected genes which play a role in amino acid metabolism, glycolysis, mitochondrial function and TCA cycle are displayed.C, DTranscript expression (C) and protein expression (D) of *HK1*, *MRPS31*, *PDHA1*, and *GOT2* (*y*‐axis) compared to transcript expression of *AXIN2* (*x*‐axis in the dotplot). Colors indicate CMS classification or normal colon tissue. Violin plots in the left panels illustrate candidate transcript and protein expression in the different CMS and tissue groups. Each datapoint corresponds to an individual tumor. Number of tumors per CMS group for (C): CMS1 ‐ 41, CMS2 ‐ 88, CMS3 ‐ 65, CMS4 ‐ 111, normal tissue ‐ 51; for (D): CMS1 ‐ 12, CMS2 ‐ 29, CMS3 ‐ 13, CMS4 ‐ 21.E, FTranscript expression (E) and protein expression (F) differences between tumor and normal colon tissue for Wnt‐high (*y*‐axis) and Wnt‐low (*x*‐axis) tumors. Colors indicate to which functional pathway each gene belongs: purple ‐ glycolysis, green ‐ mitochondrial function, pink ‐ TCA cycle and mitochondrial pyruvate metabolism.

We first studied the phenotypic differences between Wnt‐high and Wnt‐low cell lines and tumors by performing gene set enrichment analysis on the results from differential transcription and protein expression analysis (Fig 6A and Appendix Fig S5). Genes and proteins involved in mitochondrial function, respiration, and TCA cycle were expressed at higher levels in Wnt‐high compared with Wnt‐low cell lines and tumors (Fig 6A and Appendix Fig S5). In addition, we found other metabolic pathways related to cholesterol biosynthesis and metabolism of amino acids, which also partially rely on mitochondrial processes, to be upregulated on transcriptomic and in some instances also on proteomic level in Wnt‐high compared with Wnt‐low cell lines and tumors (Fig 6A and Appendix Fig S5). Differentially expressed genes showed a similar pattern of either up‐or downregulation in the two datasets (Fig 6B). The fact that the gene and protein expression changes could not only be seen in tumor tissue, but also in colorectal cancer cell lines indicates that our findings were valid to describe the actual tumor cells and were not artifacts resulting from tumor infiltration by stromal or immune cells.

Previous studies have identified the genes and proteins whose expression best correlates with metabolic activity of pathways such as TCA cycle, glycolysis, and mitochondrial maintenance (Tanner *et al*, 2018; Hartmann *et al*, 2021). We were able to confirm in our data that important regulators of glycolytic flux (*HK2*, *HK3*, *LDHA*, *GAPDH*, and *G6PD*) had lower transcript and protein levels in Wnt‐high compared with Wnt‐low tumors (Fig 6B). Genes and proteins regulating the TCA cycle (*PDHA1*, *SUCLG2*, *SUCLG1*, and *IDH2*) as well as mitochondrial function (*VDAC1*, *MRPS31*, *LARS2*, *OPA1*, and *MTIF2*) showed higher transcripts and protein levels in Wnt‐high compared with Wnt‐low tumor entities (Fig 6B). Components of the amino acid metabolism such as *SLC1A5* and *GOT2* showed a similar behavior and were higher expressed in the Wnt‐high tumor entities (Fig 6B). Our classification into Wnt‐high and Wnt‐low tumors was correlated with the expression of these metabolic genes throughout different CMS subtypes (Fig 6C and D). The tumors that were classified as CMS4 or CMS3 but belonged to the Wnt‐low tumor subgroup showed metabolic gene and protein expression levels comparable with Wnt‐low tumors classified as CMS1 (Fig 6C and D).

The transcriptomic and proteomic profiles indicated a difference in metabolic equilibrium between Wnt‐low and Wnt‐high tumors, with a more pronounced Warburg‐phenotype being observed in Wnt‐low tumors. To better understand these observations in the context of metabolic rewiring during tumorigenesis, we next compared the transcript and protein expression levels in Wnt‐high and Wnt‐low tumor samples to those of normal colon tissue (Fig 6E and F). The overall direction of expression changes was the same between tumor and normal tissue for both tumor groups. This means that despite the differences between Wnt‐high and Wnt‐low groups described in the previous paragraph (Fig 6A–D), many genes involved in glycolysis were upregulated in all tumor samples compared with normal tissue (Fig 6E and F, purple). *SLC16A1* and *PDK1*, two genes which were previously reported to be responsible for increased glycolysis upon Wnt signaling, were expressed at higher or equal levels in Wnt‐low compared with Wnt‐high tumors (Appendix Fig S6). Genes and proteins involved in mitochondrial pyruvate metabolism and TCA cycle were reduced in all tumor samples compared with normal tissue (Fig 6E and F, pink). Genes involved in mitochondrial translation were upregulated on transcript level in both tumor groups, on protein level, however, they appeared to be increased only in the Wnt‐high tumor group and not in the Wnt‐low tumor group (Fig 6E and F, green). In summary, a metabolic transcription and protein abundance switch in line with the Warburg effect was observed in both tumor types, but it was more pronounced in the Wnt‐low tumors.

**Figure 7:**
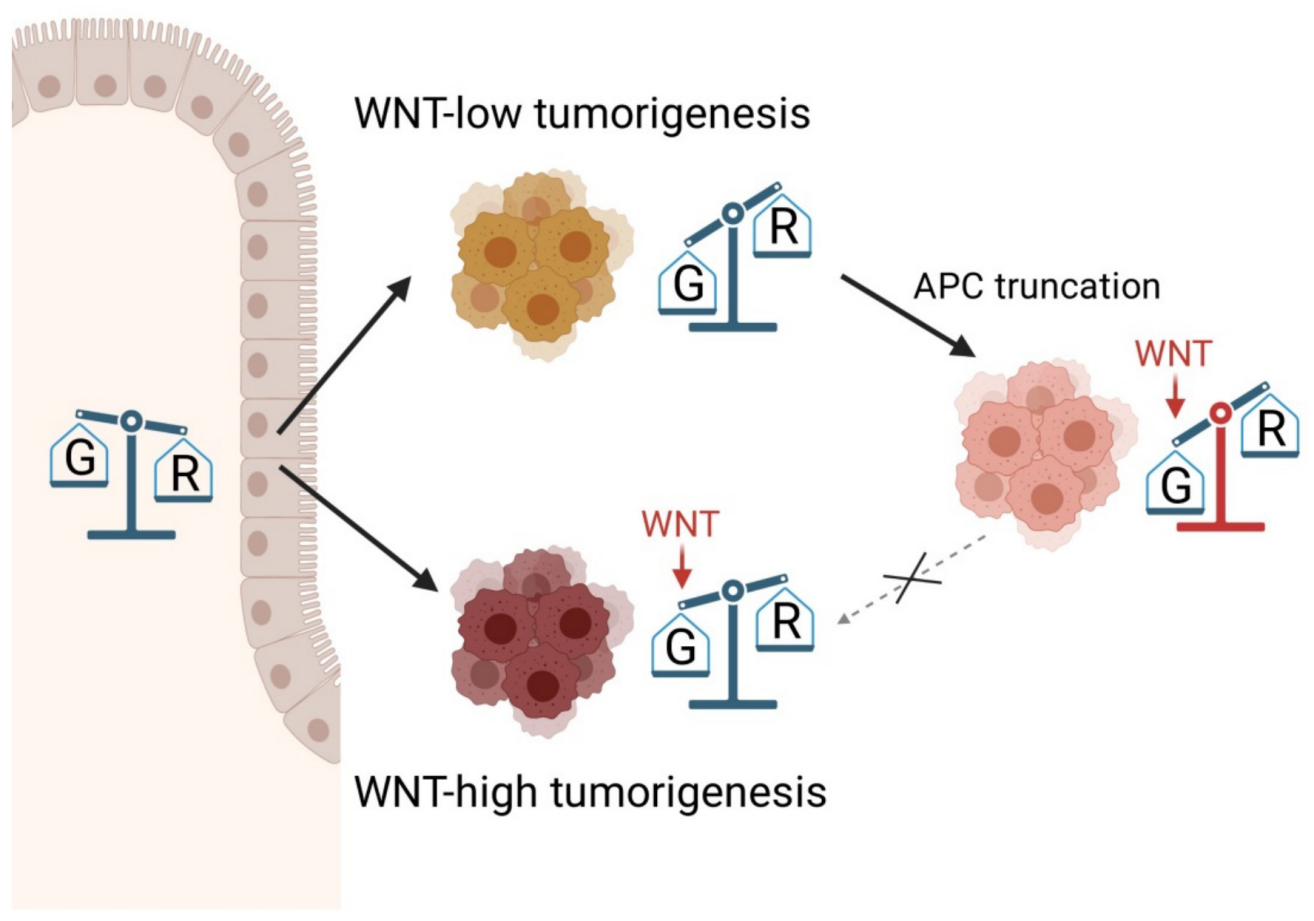
Model of metabolic balance and tumorigenesis leading to Wnt‐high and Wnt‐low colorectal cancers The balance is a schematic representation of the levels of glycolysis (G) and oxidative phosphorylation/respiration (R). Created with Biorender.com.

Our results indicate that both Wnt‐high and Wnt‐low tumors undergo a metabolic switch in accordance with the Warburg effect and tumor metabolic rewiring. However, the final metabolic equilibrium reached in both tumor types is different (Fig 7). Wnt‐dependent tumorigenesis involves a slight shift toward glycolysis, which is probably directly induced by Wnt hyperactivation, in accordance with previous studies reporting the transcriptional regulation of Warburg effect by Wnt signaling (Pate *et al*, 2014). In Wnt‐independent tumorigenesis, the shift toward glycolysis is even more pronounced. As a consequence, Wnt activation in Wnt‐low cancer cells, as modeled in our engineered APC^trunc^ RKO and HCT116 cells, leads to a metabolic imbalance because their metabolic baseline does not allow for further induction of glycolysis resulting from Wnt hyperactivation. These findings highlight that the order of genetic alterations during oncogenic transformation need to be tightly interwoven with metabolic rewiring.

## Discussion

We applied an integrative multi‐omics analysis to transcriptomic, proteomic, and functional genomic data to show that a classification of colorectal cancers into Wnt‐high and Wnt‐low entities functionally distinguishes two types of tumors. The levels of Wnt hyperactivation in each tumor type are balanced to maintain cellular fitness and a favorable metabolic state. Increasing the level of Wnt signaling, especially in those tumors that have low endogenous Wnt signaling, raises the tumor cells to an unfavorable energetic state in which their metabolic balance is perturbed. These findings showcase the context‐dependent and nonlinear effects of Wnt signaling on cellular phenotype and function during tumorigenesis.

In this study, we examined the transcriptional and protein expression differences in metabolic pathways that are essential for cellular energy supply. We showed that Wnt‐low tumors and cell lines are characterized by higher levels of genes and proteins involved in glycolysis. It has been shown in previous studies that transcript quantification and protein abundance measurements can be used to infer glycolysis pathway activity (Tanner *et al*, 2018; Hartmann *et al*, 2021). As the pathway components associated with differences in metabolic flux were higher expressed in Wnt‐low compared with Wnt‐high tumors, we inferred that glycolytic flux and lactate production is higher in Wnt‐low tumors. Genes and proteins involved in OXPHOS as well as mitochondrial function were higher expressed in Wnt‐high tumors than in Wnt‐low tumors, indicating a higher level of mitochondrial activity in Wnt‐high tumors. The overall lower expression of mitochondrial ribosomes and mitochondrial matrix components observed in our analyses could be due to an overall lower content of mitochondria linked to lower mitochondrial activity in the Wnt‐low tumor cell. In line with our interpretation of the data, previous studies have demonstrated a correlation between mitochondrial respiration and expression of specific mitochondrial components such as *OPA1* or *VDAC1* (Akkaya *et al*, 2018; Hartmann *et al*, 2021). In addition, Wnt‐high tumors also showed higher levels of genes involved in glutamine metabolism, an alternative pathway for energy supply that involves mitochondrial components (Altman *et al*, 2016). Taken together, these results indicate that the metabolism of Wnt‐high tumors in general relies more on mitochondrial function than that of Wnt‐low tumors.

Transcriptomic and proteomic differences in glycolysis and OXPHOS pathways have been previously reported for MSI tumors in comparison with MSS tumors (Vasaikar *et al*, 2019). As the Wnt‐low tumor group in our study is enriched for MSI‐H tumors, our results confirm these previous observations. Our study adds a new perspective to this observation, as we provide a link between different metabolic equilibria and dosage levels of Wnt hyperactivation during tumorigenesis. As shown in Fig 6, our classification of tumors according to their Wnt activation also allows us to predict metabolic gene and protein expression beyond the CMS classification scheme.

The metabolic phenotype of Wnt‐low tumors correlated with a higher vulnerability toward mitochondrial perturbation. Given the statistical model that we used to compare the gRNA abundances (Appendix Fig S3) as well as the successful validation using the competitive cell growth assay (Fig 5B), we are confident that this observation reflects the underlying biology and is not linked to technical artifacts that arise from differences in growth rate during the vulnerability screen. The higher vulnerability toward mitochondrial perturbation could potentially be explained by a saturation of mitochondria‐independent energy supply. As soon as mitochondria are perturbed, respiratory capacity is lost and glycolysis is used as a compensatory mechanism for energy supply. This compensatory mechanism is less efficient in Wnt‐low tumors that start with a higher baseline level of glycolysis than Wnt‐high tumors. A similar principle could serve as an explanation for increased mitochondrial vulnerability of Wnt‐low cancer cells upon APC truncation. Wnt hyperactivation in glycolysis‐performing Wnt‐low cells induced a further shift away from mitochondrial respiration toward alternative pathways as indicated by our oxygen consumption measurements. This then leads to an even higher vulnerability toward mitochondrial perturbation due to lack of non‐mitochondrial compensatory potential (Fig 7). A similar induction of mitochondrial vulnerability has been previously observed when introducing *KRAS* mutations into colorectal cancer cell lines of both Wnt‐low and Wnt‐high groups (Martin *et al*, 2017), indicating that Wnt signaling is not the only pathway important for directing metabolic rewiring in cancer. Indeed, both *APC* loss and *KRAS* mutation were shown to induce metabolic changes and accentuate glycolysis in the mouse intestine (Najumudeen *et al*, 2021).

It has been reported that Wnt signaling in Wnt‐high tumors induces Warburg effect by transcriptional activation of genes such as *MCT1* and *PDK1*, which are important regulators of glycolysis and pyruvate metabolism (Pate *et al*, 2014). The data underlying this conclusion stem from experiments conducted in Wnt‐dependent colorectal cancer cell lines under different levels of Wnt activation. When we compared the expression level of *MCT1* and *PDK1* in our tumor groups, we found that both genes and proteins were higher or equally expressed in Wnt‐low compared with Wnt‐high tumors (Appendix Fig S5). Moreover, the expression levels were also higher in normal colon tissue compared with Wnt‐high tumor tissue. This does not contradict the previous findings, as the modulation of Wnt signaling in Wnt‐high colorectal cancer cell lines does not necessarily reflect the effects of pathway activation during tumorigenesis. In fact, these data further highlight the importance of taking into account context‐dependent effects of Wnt signaling in different cell types with different metabolic equilibria.

In our study, we describe the metabolic and functional differences between Wnt‐low and Wnt‐high tumors. Depending on the cellular context and the endogenous levels of Wnt‐signaling in the initial tumor cell of origin, the sequence of oncogenic driver events leading to tumor formation may differ. In line with the idea of the serrated and the classical adenoma‐carcinoma pathway as different routes of tumorigenesis, our findings reflect that Wnt‐high and Wnt‐low tumors originate through independent mechanisms of tumorigenesis that pass through distinct scenarios of metabolic rewiring. This is indicated also by key driver mutations (*APC*, *BRAF*, and *RNF43*) and other characteristics (DNA mismatch repair proficiency, localization in the colon). Accumulation of several driver mutations that affect components outside the Wnt pathway may actually be necessary to drive Wnt‐independent colorectal cancer development (Han *et al*, 2020). Importantly, our experiments on APC^WT^ and APC^trunc^ HCT116 and RKO cell lines indicate that Wnt‐low tumors are unlikely to acquire Wnt hyperactivation, because this would drive them into an unfavorable metabolic state. Our data challenge previously formulated hypotheses about mechanisms of tumor evasion, which were built on correlating oncogenic Wnt signaling levels and immune infiltration (Luke *et al*, 2016). According to our data, if Wnt hyperactivation was a mechanism of secondary immune evasion, it would also involve substantial fitness penalties for the tumor cells. In the same lines, our study highlights that results obtained by manipulating Wnt signaling levels in cells need to be carefully evaluated when extrapolating them to general principles. The effects of Wnt hyperactivation in a cancer cell line that is in its essence Wnt‐low are different from the effects of Wnt hyperactivation during tumorigenesis leading to Wnt‐high tumors.

The exact mechanisms behind Wnt‐high as well as Wnt‐low induction of the metabolic switch will need further explorations. Previous studies have shown that the modality of Ras pathway activation may play a role in regulating glycolytic flux levels (Tanner *et al*, 2018). Depending on whether Ras activation is achieved by *KRAS* or *BRAF* mutation, and likely also dependent on the cellular context, the downstream effects of signaling may lead to different metabolic phenotypes. A recent study indicates that *BRAF* mutant tumors, which are enriched in our Wnt‐low tumor group, elicit lower levels of mitochondrial respiration than *KRAS* mutant or wild‐type tumors (Rebane‐Klemm *et al*, 2020). This, however, does not indicate whether the metabolic shift is a direct cause of *BRAF* mutation‐mediated Ras pathway activation. Along the same lines, a previous study has used quantitative assessment of pathway activation to demonstrate fundamental cell‐to‐cell heterogeneity in the modulation of Ras pathway activity after *KRAS* or *BRAF* mutation (Brandt *et al*, 2019).

Finally, a possible explanation of why Wnt‐low and Wnt‐high tumors reach different metabolic equilibria that do or do not tolerate Wnt hyperactivation could be that the cells of origin of the two tumor types are different. This cell‐of‐origin hypothesis is also consistent with the fact that Wnt‐low tumors accumulate in the proximal colon where tissue composition and development may be different than in distal colon (Wang *et al*, 2020; Fawkner‐Corbett *et al*, 2021). Indeed, the expression of genes involved in metabolism and mitochondrial function is subject to variation along the crypt‐top axis of intestinal tissue (Yang *et al*, 2016; Moor *et al*, 2018). It has been reported that the activation of Wnt signaling leads to rapid tumor development in intestinal stem cells, but not in more differentiated cells (Barker *et al*, 2009; Fessler & Medema, 2016). Differences in metabolic profile, including OXPHOS and glycolysis levels, may even identify tumors and single cells with high tumor initiation and cycling capacity (Zowada *et al*, 2021).

Our findings do not only expand our understanding of quantitative modulation of Wnt signaling during tumorigenesis, but they also showcase how large‐scale genetic perturbation data from genome‐engineered model systems can be integrated with multi‐omic data to investigate tumor heterogeneity. In this study, the detection of context‐dependent effects of Wnt signaling on cellular phenotype and function heavily relies on integrative analysis of multiple layers of molecular data, highlighting the importance of future studies addressing tumor heterogeneity at single‐cell level from a multi‐omic point of view.

## Materials and Methods

### Datasets for multi‐omic tumor profiling

The study of TCGA‐COAD and TCGA‐READ patient cohorts published by Guinney *et al* includes > 500 tumor samples for which transcriptome profiling data are available. The study by Vasaikar *et al* (2019) (CPTAC‐COAD) includes transcriptome and proteome profiling data, but only covers ~80 tumor samples. In our analysis, we thus combine the data from both studies.

### Processing of TCGA data

Clinical data and metadata concerning the experimental protocol and sequencing were accessed using the Bioconductor packages TCGAbiolinks (Colaprico *et al*, 2016) and GenomicDataCommons (preprint: Morgan & Davis, 2017). All available clinical and RNA sequencing quantification data from the TCGA‐COAD and the TCGA‐READ projects were downloaded. We used the mutation data generated from the mutest pipeline.

The downloaded RNA sequencing quantification files were assembled into one data object using the DESeqDataSetFromHTSeqCount function implemented in DESeq2 (Love *et al*, 2014). Genes with a total read count sum lower than 10 were excluded and the library size scaled counts were exported and saved for utilization in downstream analyses. To avoid batch effects in the comparison with transcriptomics data, we only used transcript quantification for samples where the sequencing was carried out at a read length of 48 base pairs (samples with 76 base pairs were excluded).

The previously generated table containing normalized sequencing counts was used as a basis for CMS classification using the original random forest classification algorithm implemented in CMSclassifier (Guinney *et al*, 2015) and default parameter settings. We only included primary tumor samples in the classification and removed normal tissue samples. In accordance with the input requirements for CMSclassifier, the normalized read counts were transformed to log2 after the addition of a pseudocount.

Wnt‐high and Wnt‐low groups were derived from k‐means clustering on RNA expression of CTNNB1 target genes using the ComplexHeatmap package (Gu *et al*, 2016). Differential gene expression between the two groups and normal tumor tissue was performed using DESeq2 (Love *et al*, 2014) based on raw sequencing counts.

### Processing of transcriptomic and proteomic data from 2019 cohort (Vasaikar *et al*, 2019; CPTAC‐COAD)

We downloaded the RNA sequencing data (RNAseq data RSEM upper‐quartile normalized, Unit: Expression (RSEM‐UQ, Log2(Val + 1))) as well as the proteomics data (Proteome data for tumor‐normal samples log‐ratio normalized, Unit: Expression (TMT, Log2ratio)) from http://linkedomics.org/cptac‐colon/, which is the link indicated in the original publication (Vasaikar *et al*, 2018, 2019). The metadata table and CMS assignment were downloaded from the supplementary material of the original publication.

Samples from the CPTAC‐COAD cohort were classified into Wnt‐low and Wnt‐high groups based on their *AXIN2* expression level. Linear models were used to estimate differential gene expression and differential protein abundance between the Wnt‐low and Wnt‐high groups. The proteomics data correspond to tumor versus normal tissue protein abundance data (logarithmic scaling). Mean relative expression levels for every gene are used in Fig 6H.

### Processing of DepMap data

Gene expression, mutation, proteomics, and gene dependency data from the cancer Dependency Map (DepMap version 19Q3; Meyers *et al*, 2017; *Ghandi et al*, 2019) was accessed using the depmap Bioconductor package (Gatto, 2020). The gene expression data correspond to the CCLE project RNAseq transcripts per million (TPM) for protein coding genes only (scaled as log2(TMP + 1)). Mutation data correspond to merged mutation calls (coding region and germline filtered) from the CCLE project. Proteomics data correspond to normalized protein abundance from quantitative proteome profiling by mass spectrometry (Nusinow *et al*, 2020). The gene dependency data correspond to the batch corrected CERES inferred gene effects that were derived from whole‐genome CRISPR‐Cas9 knockout viability screens.

Wnt‐high and ‐low groups identified by clustering of TCGA 2015 samples correlated well with the expression of *AXIN2*, this is why we were able to use *AXIN2* expression to identify Wnt‐high and Wnt‐low cell lines in the DepMap data. Linear models were used to estimate differential gene expression, differential protein abundance and differential gene dependence between the Wnt‐low and Wnt‐high groups (Smyth, 2011).

### Gene identifier conversion

The conversion of gene identifiers between ENSEMBL ids, ENTREZ ids and gene symbols was performed using the bitr function of the Bioconductor package clusterProfiler (Yu *et al*, 2012). Nonunique mapping genes or genes without mapping were excluded from the analysis.

### Gene set enrichment analysis

Gene set enrichment analysis was performed using gene lists ordered according to the statistic provided by either DESeq2 or linear model. For Fig 2, gene set enrichment analysis was performed for the MSigDB hallmark gene set HALLMARK_WNT_BETA_CATENIN_SIGNALING (Subramanian *et al*, 2005; Liberzon *et al*, 2015). For Figs 3, 4, and 6, gene set enrichment analysis was performed for (i) Reactome pathway annotation (using the gsePathway() function of ReactomePA package; Yu & He, 2016) and (ii) MSigDB hallmark gene sets and GO term gene sets of the “Cellular component” ontology (using msigdb package for gene set annotation and GSEA() function from ClusterProfiler package (Yu *et al*, 2012) for testing). The packages implement multiple‐testing correction using the Benjamini–Hochberg method. Adjusted *P*‐value cutoffs (typically 0.05) are indicated in the figure legends.

### R package versions

R version 4.1.3, enrichplot_1.14.2, data.table_1.14.2, circlize_0.4.14, ComplexHeatmap_2.10.0, gscreend_1.1.0, GenomicDataCommons_1.18.0, magrittr_2.0.2, TCGAbiolinks_2.22.4, limma_3.50.1, DESeq2_1.34.0, SummarizedExperiment_1.24.0, MatrixGenerics_1.6.0, matrixStats_0.61.0, GenomicRanges_1.46.1, GenomeInfoDb_1.30.1, ReactomePA_1.38.0, msigdbr_7.4.1, ggrepel_0.9.1, org.Hs.eg.db_3.14.0, AnnotationDbi_1.56.2, IRanges_2.28.0, S4Vectors_0.32.3, Biobase_2.54.0, BiocGenerics_0.40.0, clusterProfiler_4.2.2, depmap_1.8.0, cowplot_1.1.1, pheatmap_1.0.12, ggplotify_0.1.0, patchwork_1.1.1, forcats_0.5.1, stringr_1.4.0, dplyr_1.0.8, purrr_0.3.4, readr_2.1.2, tidyr_1.2.0, tibble_3.1.6, ggplot2_3.3.5, tidyverse_1.3.1.

### Cell lines and culture

HCT116 cells were cultured in McCoy's medium (Life Technologies). RKO cells were cultured in DMEM medium (Life Technologies). All media were supplemented with 10% fetal calf serum (PAA). Cell lines were obtained from ATCC and authentication of genotypes was performed by SNP profiling (Multiplexion, Heidelberg). The absence of mycoplasma infection was confirmed by regular testing.

### Generation and validation of RKO and HCT116 cell lines with truncated APC

The generation of RKO and HCT116 cell lines with truncated APC is the same approach as described in Zhan *et al* (2019). Cell line RKO APC^trunc^#5 corresponds to the RKO APC truncated cell line clone #5 described in Zhan *et al* (2019).

The sgRNA targeting the APC gene was designed using the E‐CRISP sgRNA design tool (Heigwer *et al*, 2014). The sequenco of the designed sgRNA was: sgAPC 5′‐TCTGCTGGATTTGGTTCTA**GGG** – 3′ (bold letters indicate PAM sequence). Pairs of oligonucleotides encoding the sgRNA were synthesized by Eurofins Inc. Oligonucleotides were phosphorylated, annealed, and cloned into a Bbs1 digested px459 plasmid (#62988, Addgene) using Quick Ligase (NEB). To generate an APC truncation, RKO and HCT116 cells were transiently transfected with 2 μg of px459 with sgAPC. After 48 h, cells were selected with 1 μg/ml of puromycin for 48–72 h. Single clones were generated by serial dilutions in 96‐well plates. After 10–15 days, colonies derived from single clones were expanded for further analyses. Targeted deep sequencing of PCR amplified APC genomic locus was performed to assess indel formation introduced by the sgRNA. Genomic DNA of APC mutant single‐cell clones was isolated using DNeasy Blood and Tissue Kit (Qiagen). Primer pairs were designed 100–150 bp up and downstream of the sgRNA targeting site and adapters were added during the second step of the nested PCR. The PCR primers for the first PCR step were 5′‐TCCCTACACGACGctcttccgatctTCAGACGACACAGGAAGC‐3′ and 5′‐AGTTCAGACGTGTGctcttccgatctACATAGTGTTCAGGTGGACT‐3′. The resulting PCR products were purified with the PCR Clean‐up Kit (Machery‐Nagel) and amplified with a second PCR step to introduce unique indexes. The second PCR was purified using Agencourt Ampure XP Beads (Beckman Coulter), and samples were sequenced on a MiSeq (Illumina) by the Genomics and Proteomics Core Facility of the DKFZ. The multiple sequence alignment tool ClustalOmega (Sievers *et al*, 2011) was used to analyze indel formation. The two single‐cell clones used in this study were selected based on successful APC truncation and Wnt/*TCF4*‐reporter activity.

### *TCF4*/Wnt‐reporter assay

The luciferase‐based dual Wnt reporter assay was performed as described previously (Demir *et al*, 2013). In brief, cells were seeded in a white, flat‐bottom 384‐ or 96‐well plates. Twenty‐four hours later, cells were transfected with a plasmid encoding tha firefly luciferase under control of a promoter composed by repeats of the TCF4‐binding sites and with a control plasmid encoding renilla luciferase under control of a CMV promoter. Dual‐luciferase readout was performed 48 h after transfection using Mitras LB940 plate reader (Berthold Technologies). The firefly luciferase signal was normalized to the renilla luciferase signal. For RKO three‐independent experiments were performed in total. For HCT116 four‐independent experiments confirmed these results.

### Microarray analysis

We performed microarray‐based gene expression analysis on RKO wild‐type and the isogenic APC^trunc^ clone (RKO APC^trunc^#5) to identify genes that are differentially expressed between the two cell lines. Two independent replicates for both RKO APC^WT^ and RKO APC^trunc^ were performed. RNA from cell pellets was extracted using the Quiagen RNeasy kit and RNA quality assessed using the Bioanalyzer Eukaryote Total RNA Pico assay. For microarray measurement, Affy Human U133Plus 2.0 chip was used in combination with the iScan array scanner. Data analysis was performed according to the Bioconductor workflow vignette for Affymetrix microarrays (Klaus & Reisenauer, 2016).

### CRISPR screening

The 90 k Toronto human Knockout pooled library (TKO) was a gift from Dr. Jason Moffat (1000000069, Addgene; Hart *et al*, 2015). The plasmid library was amplified using ElectroMAXTM Stbl4TM cells (Invitrogen) and transfected into HEK293T cells (ATCC) with TransIT‐LT1 (Mirus Bio) transfection reagent along with psPAX2 (12260, Addgene) and pMD2.G (12259, Addgene) packaging plasmids for production of lentivirus.

HCT116, RKO, HCT116 APC^trunc^#2 and RKO APC^trunc^#5 cells stably expressing Cas9 (73310, Addgene) were transduced with the previously generated virus in the presence of 8 μg/ml polybrene (Merck Millipore). The multiplicity of infection (MOI) was equal to 0.3 and each gRNA was present in 500 cells on average. The day after, puromycin‐containing medium was added and the cells cultures for 48 h in this selection medium. The cells were then grown in medium without puromycin for 12 doubling times and split every 3 days at a coverage of 500× (each gRNA was present in 500 cells on average). The time needed for each cell line to accomplish 12 doublings was calculated based on doubling times previously estimated from counting cells over a defined period of time. After 12 doubling times, time point T1 was collected by collecting and pelletting the cell pool. Genomic DNA from collected cells was extracted using QIAamp DNA Blood Maxi kit (Qiagen).

To amplify and quantify the gRNA sequences in the plasmid library and at time point T1, many PCRs were performed, each using 1 μg of genomic or plasmid library DNA, Q5 Hot Start HF polymerase (NEB), and primers harboring the Illumina TruSeq adapter sequences. The number of PCRs for each sample was such that each gRNA in the library pool was represented on average 250 times in the total amount of DNA used for PCRs. PCR products were purified using DNA Clean and Concentrator TM‐100 (Zymo Research) and MagSi‐NGSprep Plus beads (Steinbrenner). DNA concentrations of the purified PCR products were measured using Qubit HS DNA Assay (Thermo Fisher). The amplicon size in the PCR products was verified using DNA High Sensitivity Assay on a BioAnalyzer 2100 (Agilent). Finally, the libraries were sequenced on a NextSeq (Illumina) sequencer with a 75 bp single‐end protocol and addition of 25% PhiX control v3 (Illumina).

### Statistical analysis

gRNAs were counted from the raw sequencing files using the count function with automatic sequence trimming provided by MAGeCK (Li *et al*, 2014). The gRNA abundances were quantified in the plasmid library and in the four cell pools at time point T1.

Differential gene essentiality was calculated using the gscreend package (Imkeller *et al*, 2020) and comparing normalized gRNA abundances at time point T1 in the APC^WT^ cell pool (*c*_*T1*,*WT*_) versus APC^trunc^ cell pool (*c*_*T1*,*APC*_). Values indicated as differential gene essentiality correspond to log2(*c*_*T1*,*APC*_/*c*_*T1*,*WT*_). Logarithmic fold changes at gRNA level were calculated individually for every cell line as log2(*c*_*T1*_/*c*_*plasmid*_), where *c*_*T1*_ denotes the normalized gRNA count at T1 and *c*_*plasmid*_ the normalized gRNA count in the plasmid library (normalized counts correspond to counts scaled to the total number of gRNA counts in the sequencing library).

### Competitive cell growth assay with LARS2 knockout

gRNAs used for validation experiments around *LARS2* knockout were selected from the set of *LARS2* targeting guide RNAs present in the TKO library. The sequence of the two selected gRNAs was (bold letters indicate PAM sequence):

LARS2_1: 5′‐ CGTTGGCAGACCTTCCAGAA ‐3’.

LARS2_2: 5′‐ CCCTATCCCAGCTGAAACAC ‐3′.

For fluorescent labeling of control knockout cells, we used a lentiviral vector (lentiCRISPRv2) encoding eGFP and an sgRNA targeting *AAVS1* (pLenti_HDlib_sgRNANOonChip_Puromut_eGFP_AAVS1). For fluorescent labeling of *LARS2* knockout cells we used two lentiviral vectors encoding mScarlet and each one of the above described sgRNA targeting *LARS2* (pLenti_HCLib_NoC_mScarlet_LARS2_1 and pLenti_HCLib_NoC_mScarlet_LARS2_2).

The isolated lentiviral plasmids pLenti_HCLib_NoC_mScarlet_LARS2_1, pLenti_HCLib_NoC_mScarlet_LARS2_2 and pLenti_HDlib_sgRNANOonChip_Puromut_eGFP_AAVS1 were transfected into HEK293T cells (ATCC) with TransIT‐LT1 (Mirus Bio) transfection reagent along with psPAX2 (12260, Addgene) and pMD2.G (12259, Addgene) packaging plasmids to produce lentivirus. HCT116 and RKO cells (WT and APC truncated) were transduced with the viruses. Puromycin was added after 24‐h incubation to select for transduced cells. The next day, the cell lines were pooled in the following manner: HCT116‐WT with stable knockout in *LARS2* and expression of *mScarlet* were mixed 1:1 with HCT116‐WT, in which *AAVS1* was targeted and *eGFP* expressed. The same pooling was performed for HCT116‐APC and both RKO lines.

The cell pools were grown for 2 weeks and the amount of eGFP and mScarlet expressing cells in each pool was quantified using FACS analysis at different time points (after 0, 3, 5, 7, 10, 12 and 15 days after pooling). FACS analysis was made with LSR Fortessa (BD Biosciences) and FlowJo v10.1 software.

### Measurement of basal respiration

For the respiration measurements, knockout of *LARS2* was performed using lentiviral vectors encoding each of the above‐described LARS2 targeting gRNAs (plcKO_Wu_LARS2_1 and plcKO_Wu_LARS2_2). Generation of lentivirus and transduction of cell lines was performed as described above. The basal respiration rate was measured 5–7 days after transduction using Agilent Seahorse Instrument in combination with different drug treatment. A day prior to the analysis, untreated HCT116 and RKO cells as well as cells transduced with either *LARS2* or *AAVS1* targeting sgRNAs were seeded in provided 96‐well plates. In the experiments involving Wnt3a treatment, 100 ng/ml recombinant Wnt3a was added to the cell culture medium 24 h prior to Seahorse measurement. The cell number was adjusted to 20,000 cells per well. Furthermore, Seahorse XF Calibrant was left for incubation at 37°C overnight without CO_2_ supply and the Agilent Seahorse XFe96 Sensor Cartridge was hydrated as described in the protocol.

On the day of analysis, the sensor cartridge was transferred in pre‐warmed XF Calibrant. In addition, seeded cells were washed two times with preprepared Seahorse XF DMEM Medium containing 1 mM pyruvate, 2 mM L‐glutamine and 10 mM glucose. The plate was then incubated at 37°C without CO_2_ for 1 h prior to the analysis. The analysis was performed by Seahorse Wave Desktop Software (Agilent). The oxygen consumption measurements used in this study are three measurements A before the addition of any drug (basal respiration + non‐mitochondrial oxygen consumption) and three measurements B after the addition of 1 μM Rotenone and 1 μM Antimycin A (non‐mitochondrial oxygen consumption). The basal respiration rate was calculated as the difference between the mean of measurements A minus the mean of measurements B. To quantify protein content, the cells were finally lysed with 25 μl RIPA lysis buffer containing appropriate amounts of protease inhibitor. BCA assay Pierce™ BCA Protein Assay Kit was used to determine protein content, which was subsequently used to normalize the oxygen consumption data of the Mito Stress Test.

## Author contributions

**Katharina Imkeller:** Conceptualization; data curation; formal analysis; supervision; investigation; visualization; methodology; writing – original draft; writing – review and editing. **Giulia Ambrosi:** Validation; investigation. **Nancy Klemm:** Investigation. **Ainara Claveras Cabezudo:** Investigation. **Luisa Henkel:** Validation; investigation. **Wolfgang Huber:** Conceptualization; supervision; writing – review and editing. **Michael Boutros:** Conceptualization; supervision; writing – original draft; writing – review and editing.

In addition to the CRediT author contributions listed above, the contributions in detail are:

KI performed the computational analyses on tumor and DepMap data, analyzed the data from the screening and validation experiments. GA generated and validated the cell lines with APC truncation and performed the screening experiment. NK performed and analyzed the data from the validation experiments. ACC wrote R code for the analysis of TCGA datasets. GA and LH guided the validation experiments. MB and WH designed and supervised the research. KI and MB wrote the paper. All authors read and approved the final manuscript.

## Disclosure and competing interests statement

The authors declare that they have no conflict of interest.

## Supporting information




Appendix

## Data Availability

Raw gRNA counts of the CRISPR screen performed in the context of this study can be downloaded from https://github.com/boutroslab/Supp_Imkeller_2021/blob/main/external_data/CRISPR_screen_gRNA_counts.csv.gz. All other public datasets used in the study can be accessed as described in the Materials and Methods section and in the corresponding rmarkdown files. All unique biological materials are available from the authors upon request. The code used for analysis and visualization is deposited at github.com/boutroslab/Supp_Imkeller_2021. The README file indicated which script is used for which analysis step.
