# Supplementary material for: Metabolic balance in colorectal cancer is maintained by optimal Wnt signaling levels
Source: Mol Syst Biol. 2022 Jul 29;18(8):e10874. doi: 10.15252/msb.202110874 (PMC9336172; doi:10.15252/msb.202110874)

Appendix

Metabolic balance in colorectal cancer is maintained by optimal Wnt signaling levels

Katharina Imkeller, Giulia Ambrosi, Nancy Klemm,
Ainara Claveras Cabezudo, Luisa Henkel, Wolfgang Huber, Michael Boutros

Table of content:

- Appendix Figure S1: Contribution of Wnt target genes to differences between tumor and normal tissue
- Appendix Figure S2: Engineered APC truncation in HCT116 and RKO cells
- Appendix Figure S3: Analysis of whole-genome CRISPR screen
- Appendix Figure S4: Gene set enrichment analysis on genetic dependencies
- Appendix Figure S5: Gene set enrichment analysis on differential transcript and protein expression
- Appendix Figure S6: Transcript expression and protein expression of *SLC16A1* and *PDK1*

Appendix Figure S1

Contribution of Wnt target genes to differences between tumor and normal tissue
A: PCA representation of tumor and normal tissue samples from TCGA-COAD and TCGA-READ cohorts. The PCA is calculated based on expression of CTNNB1 target genes (MsigDb pathway HALLMARK_WNT_BETA_CATENIN_SIGNALING). CMS classification as in Figure 1 is represented in color.
B: Principal component scores of the most important 15 genes for PC1 and PC2. These scores correspond to the projection of the original expression data on the directions of the principal component. They were calculated using the R function prcomp.
C-E: *NKD1*, *TCF7* and *WNT5B* RNA expression in TCGA samples classified into the different CMS as well as normal colon tissue.

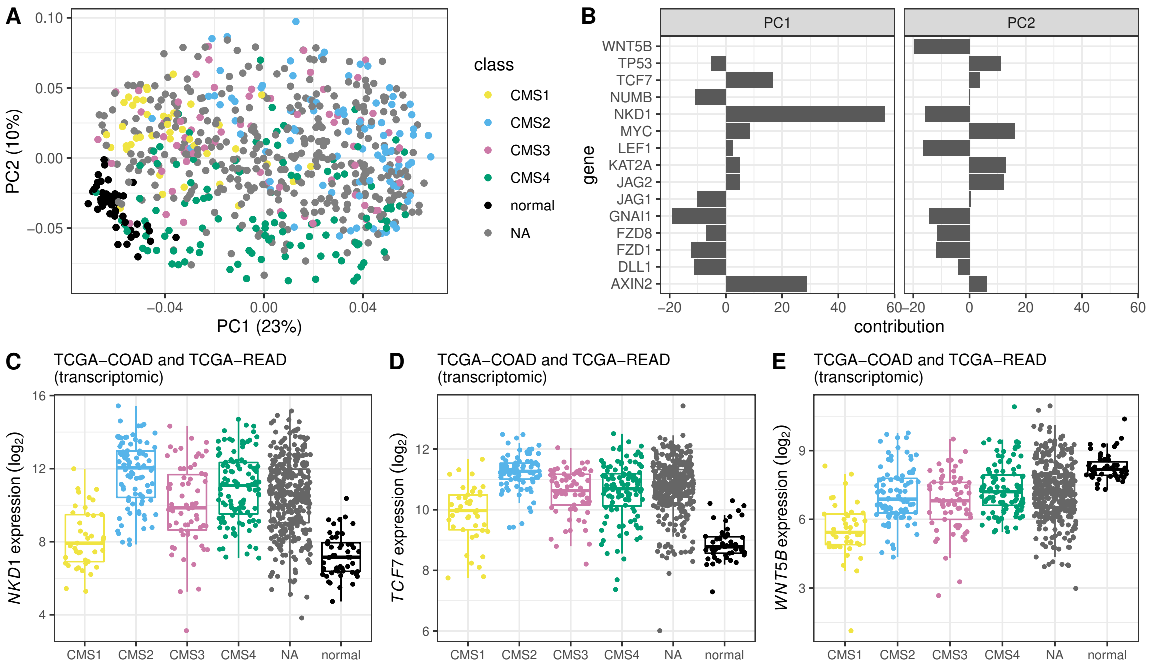

Appendix Figure S2

Engineered APC truncation in HCT116 and RKO cells
A: Strategy for generation of APC^{trunc} single cell clones from HCT116 and RKO cell lines
B: Western blot analysis of APC protein size and expression in HCT116, HCT116-APC^{trunc}#2, RKO and RKO-APC^{trunc}#5 cells. Detection of viculin was used as a loading control.
C: TCF4/Wnt-reporter activity in HCT116, HCT116-APC^{trunc}#2, RKO and RKO-APC^{trunc}#5 cells. Results show normalized mean +/- SD reporter activity over seven technical replicates within one experiment. The data is representative of at least three independent experiments that were performed.

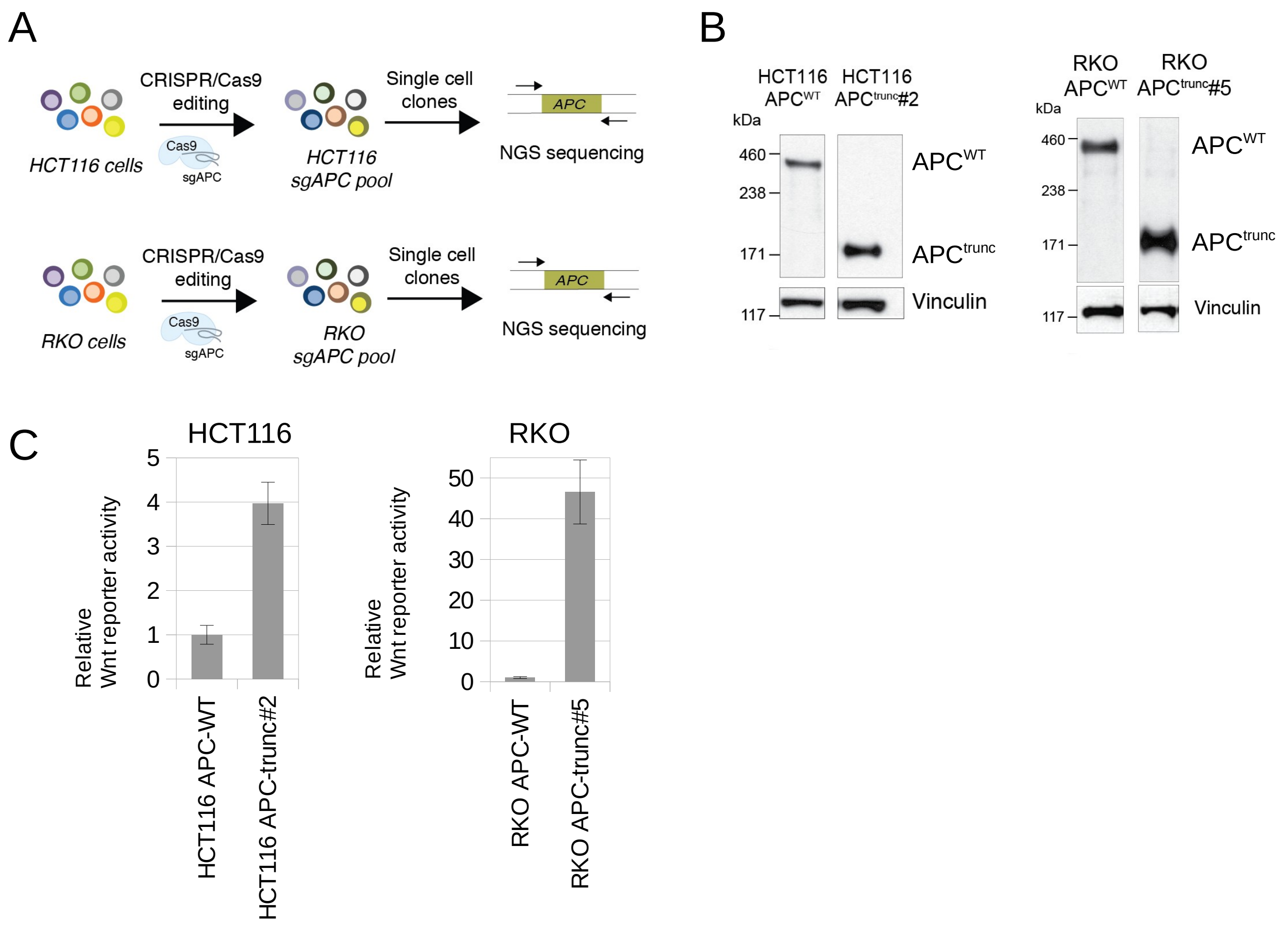

Appendix Figure S3

Analysis of whole-genome CRISPR screen.

A: Between replicate correlation coefficient for the four different cell lines (HCT116 and RKO with APC^{WT} ("WT") and APC^{trunc} ("APC"). Each screen was performed in 2 replicates.

B: Between replicate correlation. gRNA counts after the screen in replicates R2 vs. R1 are displayed (pseudocount added and displayed as logarithm of 2). The red line indicates the results of a linear model fitting (using the function ggplot2::geom_smooth(method = "lm")).

C: Precision-recall curves based on "core-essential" and "non-essential" gene assignment as in BAGEL software (Hart et al. 2016). One precision-recall curve was calculated for every individual cell line by comparing gRNA abundance before and after the screen. All four screens showed satisfying precision-recall statistics, indicating a good data quality for phenotype detection.

D: Results from gscreend analysis comparing gRNA abundance after the screen in APC^{trunc} versus APC^{WT} cells. Genes are classified as "core-essential" or "non-essential" as in C. The horizontal line indicates an adjusted p-value of 0.1.

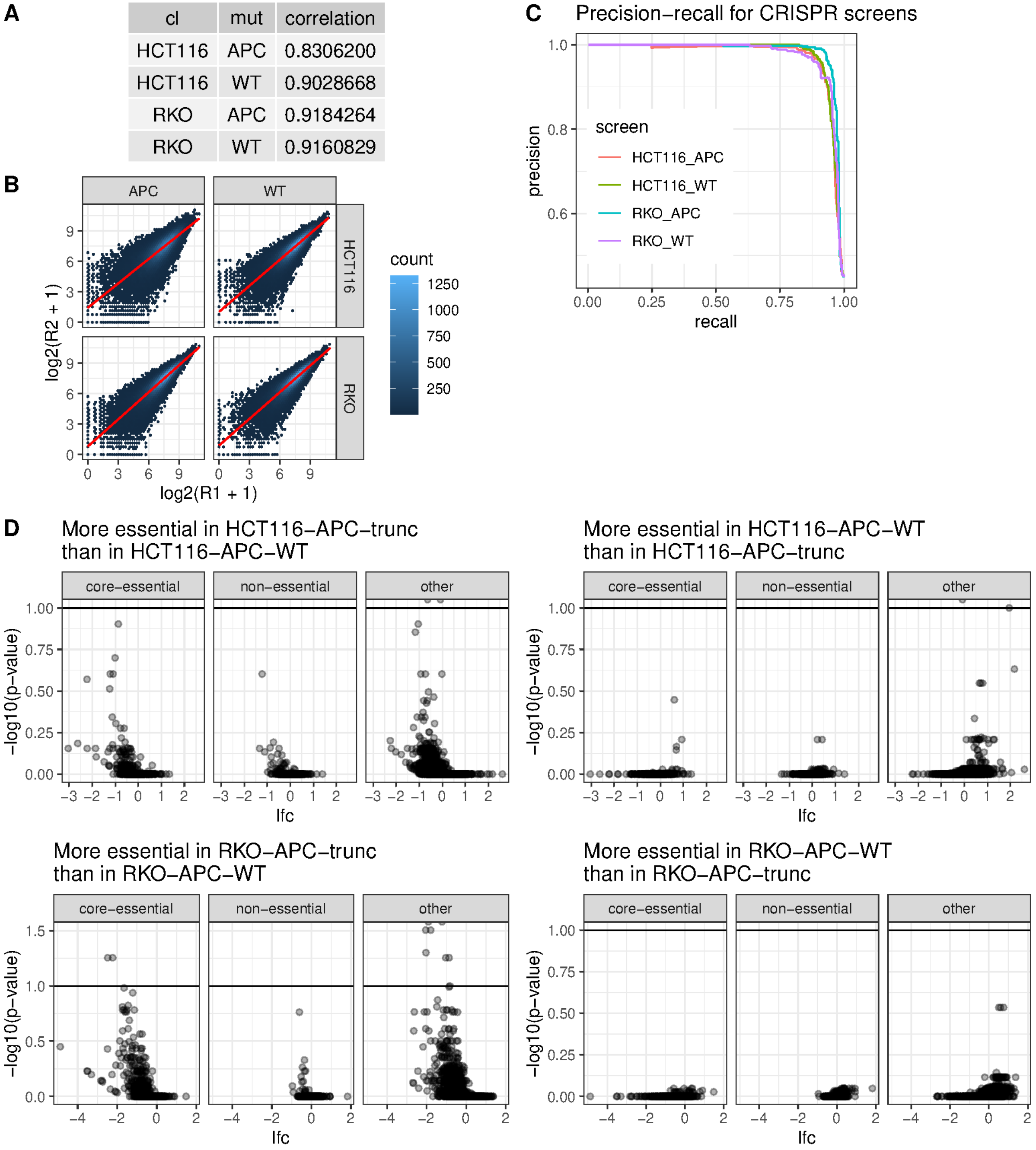

cl	mut	correlation
HCT116	APC	0.8306200
HCT116	WT	0.9028668
RKO	APC	0.9184264
RKO	WT	0.9160829

Appendix Figure S4

Gene set enrichment analysis on genetic dependencies.
We performed gene set enrichment analysis using Reactome (A-C) and GO cellular component (D-F) gene set annotation. For both annotation schemes, enrichment analysis was performed for RKO background (A+D), HCT116 background (B+E) and DepMap data (C+F). For RKO and HCT116 background the genes were ordered according to their differential essentiality in APC^{trunc} compared to APC^{WT} cells. For the analysis on DepMap data, genes were ordered according to their differential essentiality in Wnt-high compared to Wnt-low cell lines. Negative enrichment scores thus indicate gene sets whose members are more essential in APC^{trunc} or Wnt-high cells than in APC^{WT} or Wnt-low cells.

S4A) Reactome GSEA, differential gene dependency (RKO APC^{trunc} vs. APC^{WT})

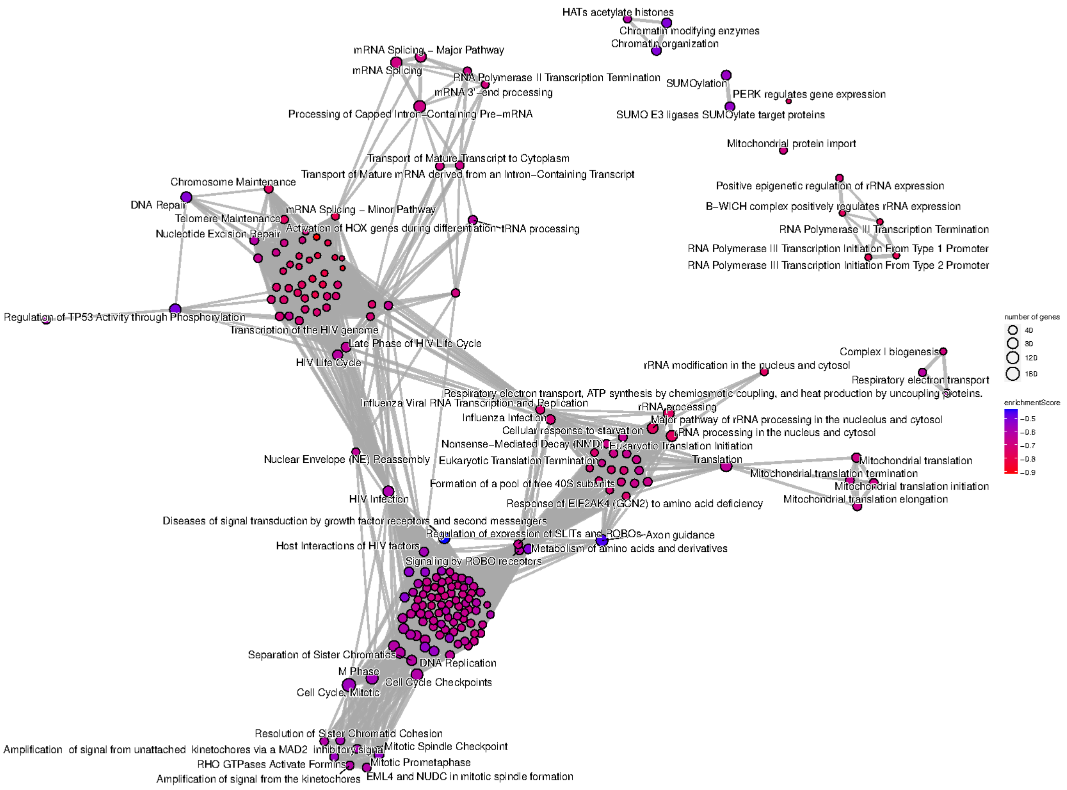

S4B) Reactome GSEA, differential gene dependency (HCT116 APC^{trunc} vs. APC^{WT})

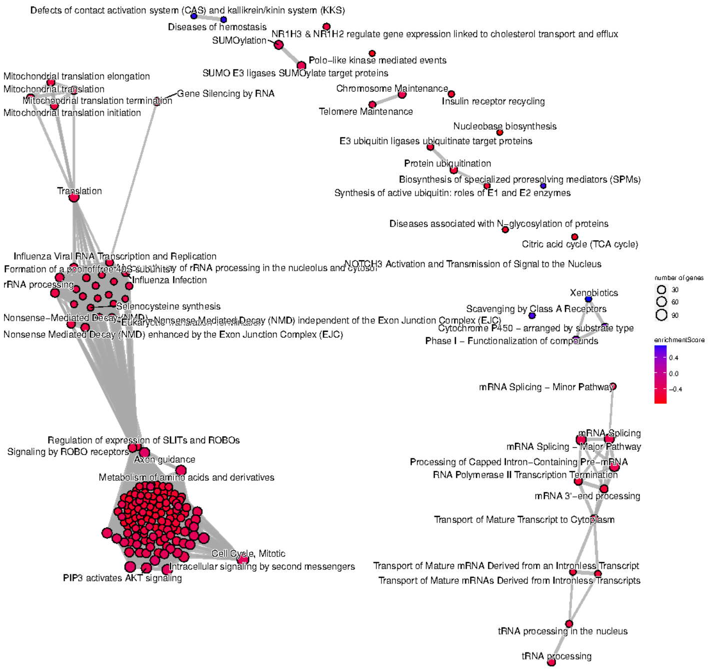

S4C) Reactome GSEA, differential gene dependency (DepMap Wnt-high vs. Wnt-low)

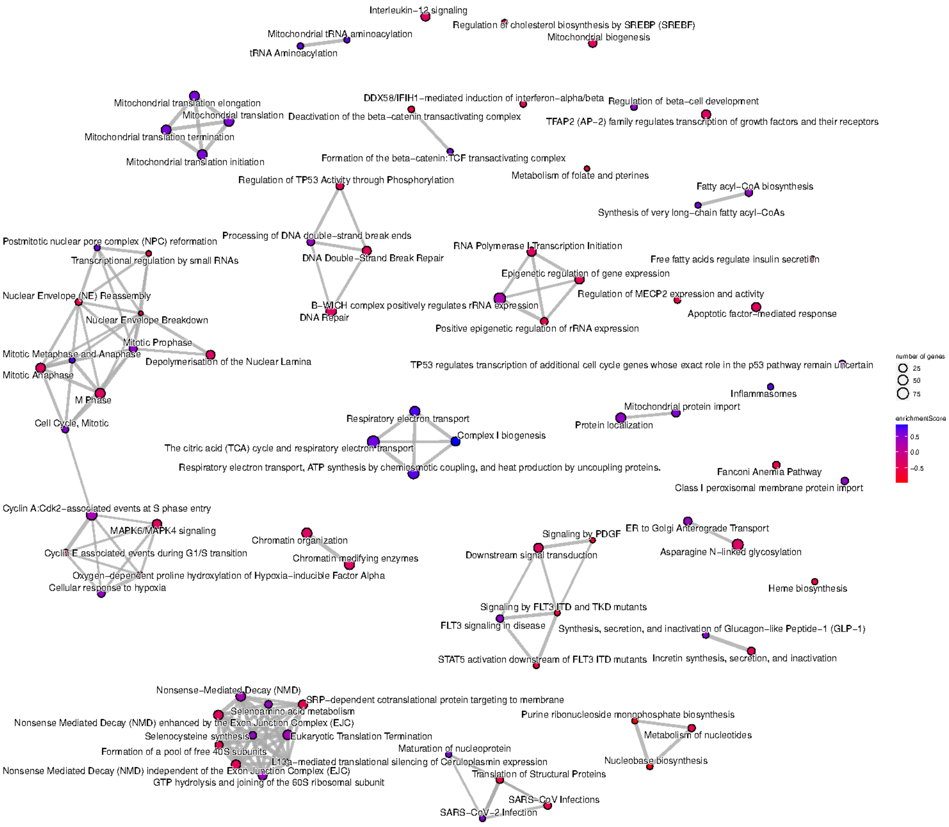

S4D) GO cellular component GSEA, differential gene dependency (RKO APC^{trunc} vs. APC^{WT})

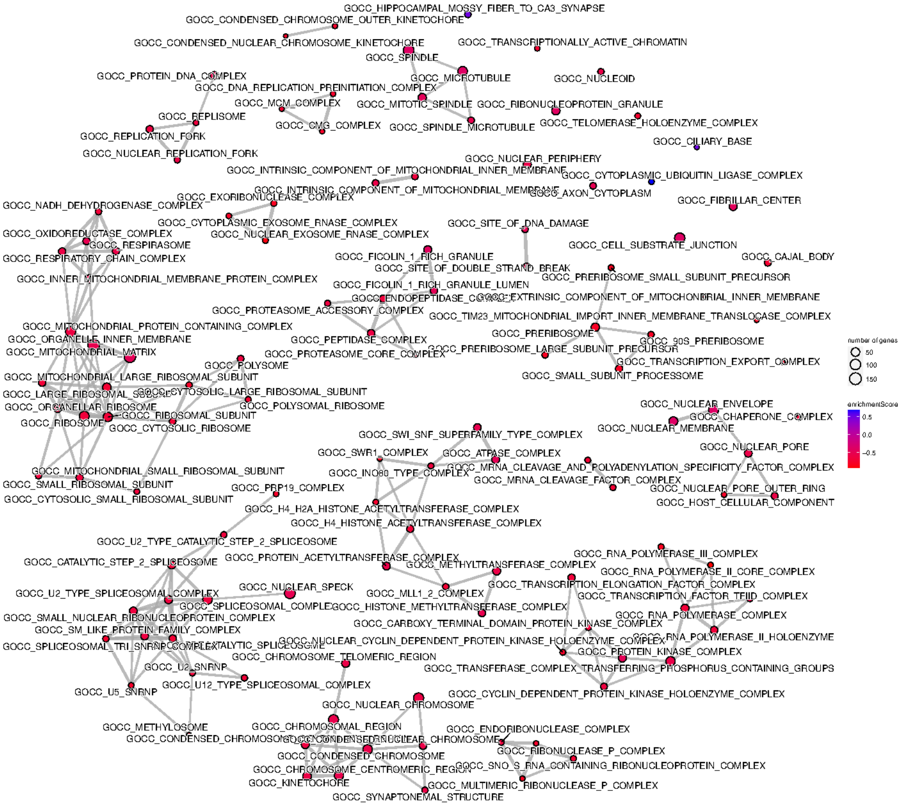

S4E) GO cellular component GSEA, differential gene dependency (HCT116 APC^{trunc} vs. APC^{WT})

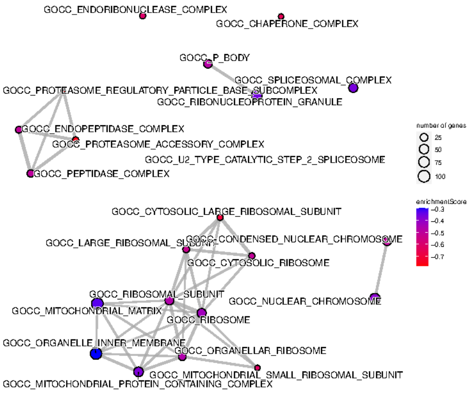

S4F) GO cellular component GSEA, differential gene dependency (DepMap Wnt-high vs. Wnt-low)

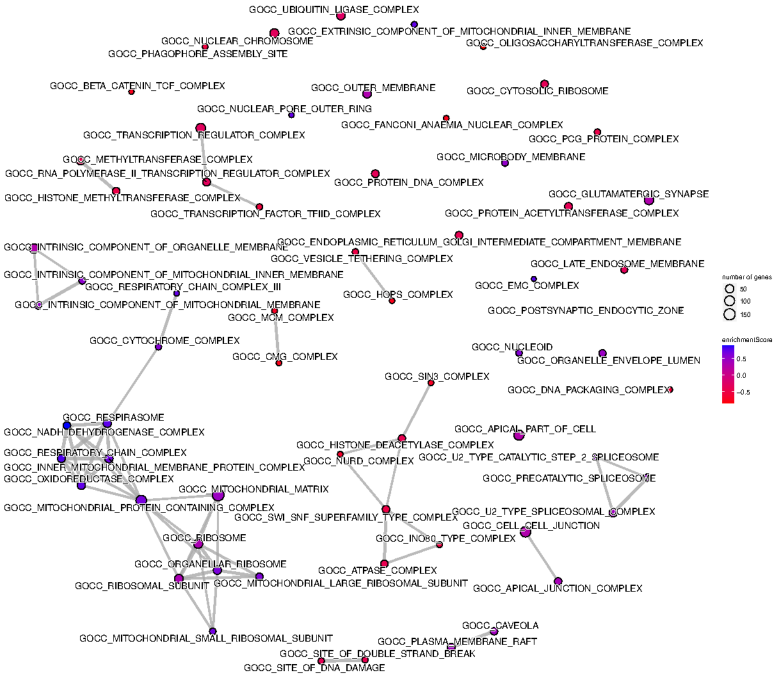

Appendix Figure S5

Gene set enrichment analysis on differential transcript and protein expression.
We performed gene set enrichment analysis using Reactome (A+B and E+F) and GO cellular component (C+D and G+H) gene set annotation. For both annotation schemes, enrichment analysis was performed for tumor tissue (TCGA, A-D) and colorectal cancer cell lines (DepMap, E-H). The gene list used for GSEA was ordered according to differential transcript expression (A, C, E, G) or differential protein abundance (B, D, F, H) in Wnt-high compared to Wnt-low tumors and cell lines. Positive enrichment scores indicate gene sets whose members are higher expressed in Wnt-high compared to Wnt-low cells and tumors.

S5A) Reactome GSEA, TCGA transcriptomic (Wnt-high vs. Wnt-low)

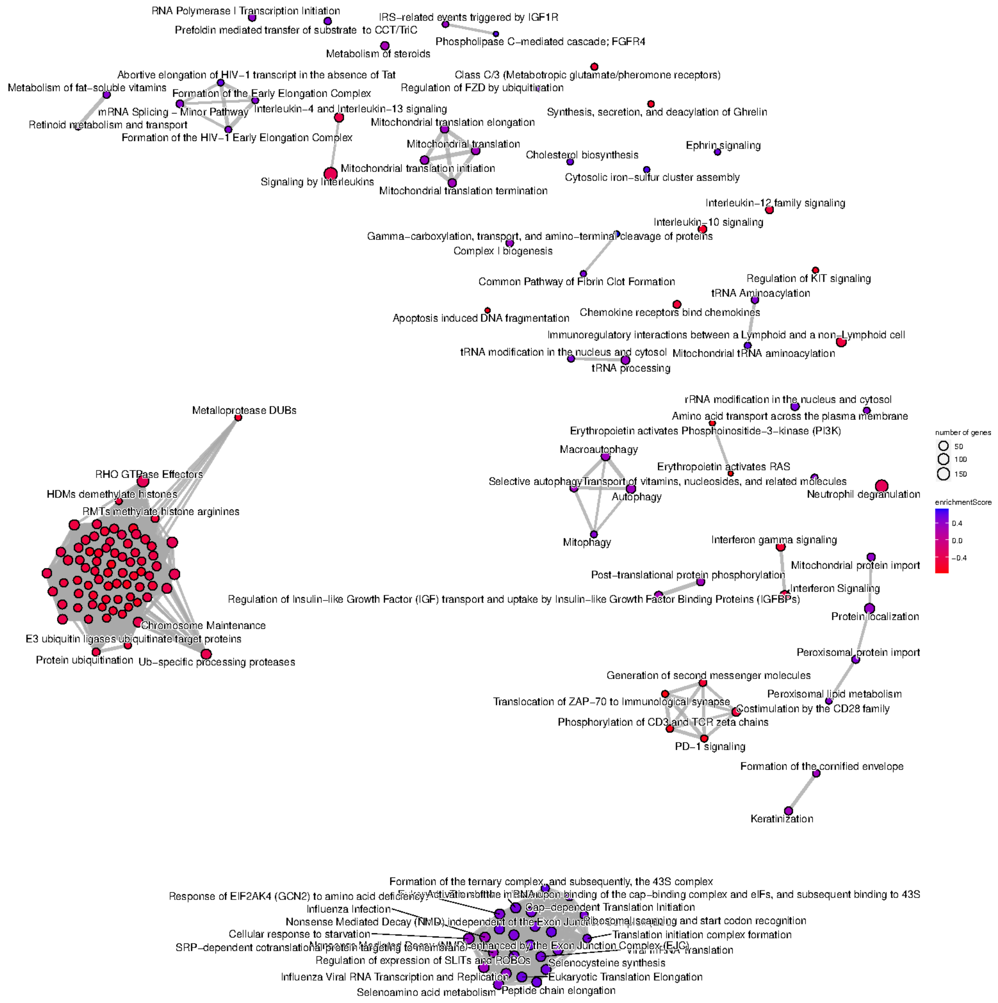

S5B) Reactome GSEA, TCGA proteomic (Wnt-high vs. Wnt-low)

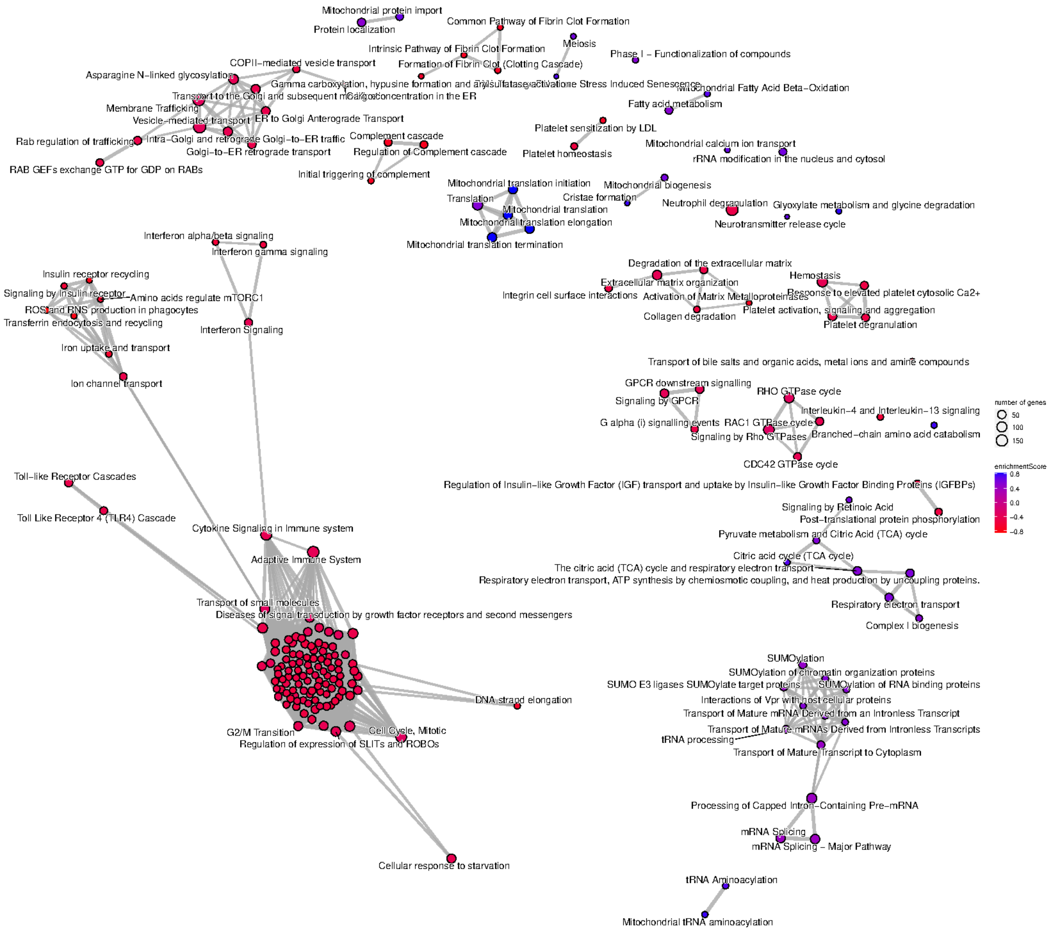

S5C) GO cellular component GSEA, TCGA transcriptomic (Wnt-high vs. Wnt-low)

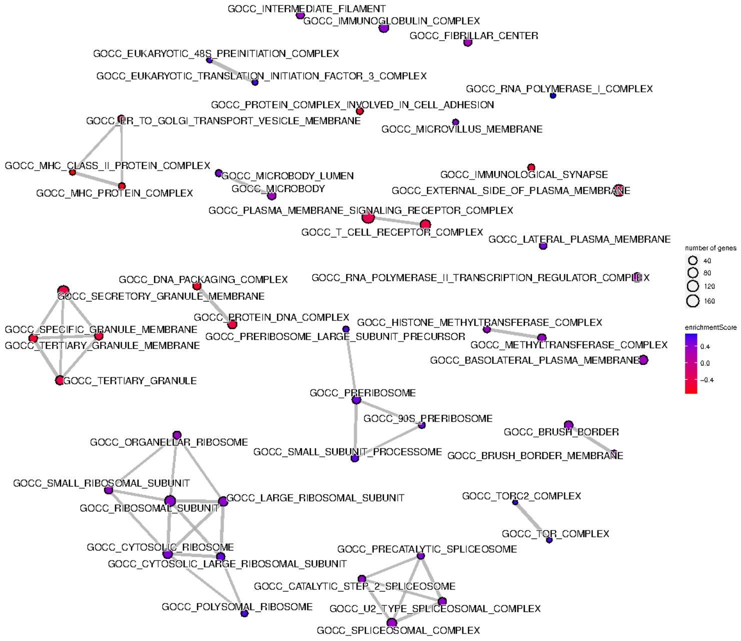

S5D) GO cellular component GSEA, TCGA proteomic (Wnt-high vs. Wnt-low)

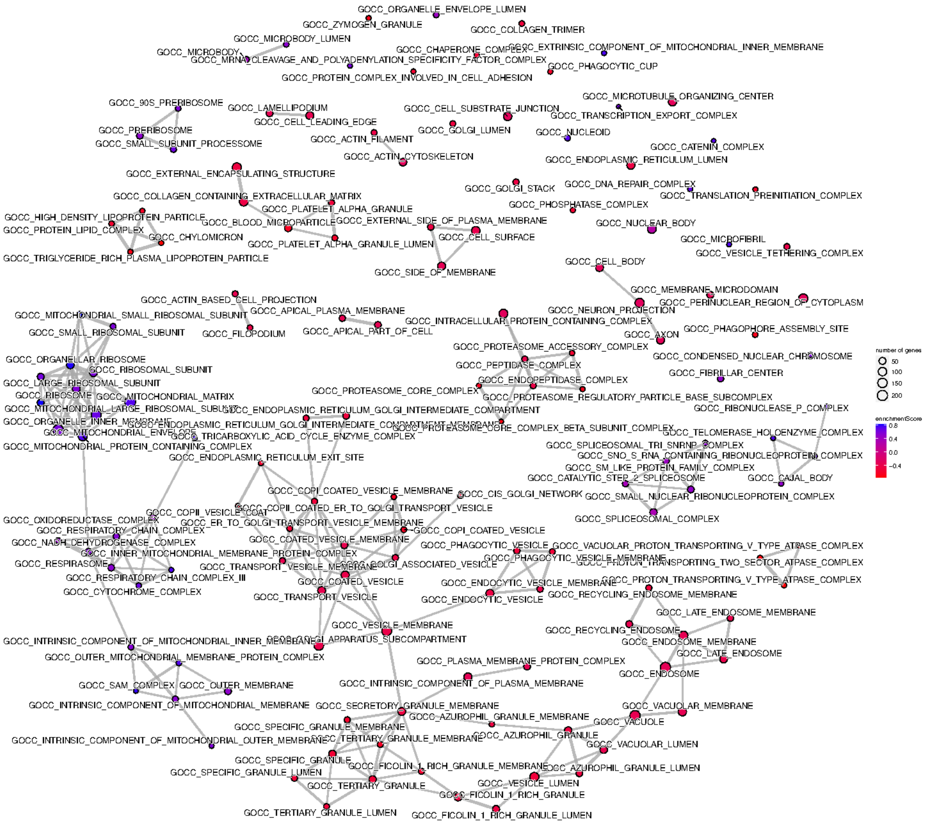

S5E) Reactome GSEA, DepMap transcriptomic (Wnt-high vs. Wnt-low)

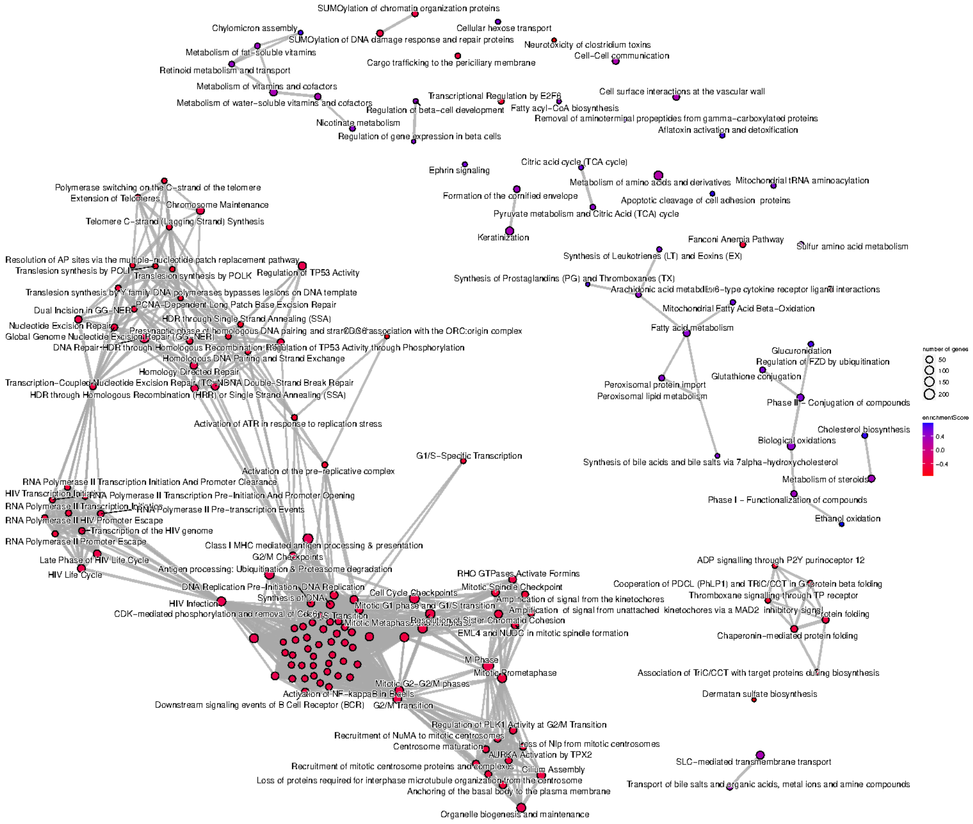

S5F) Reactome GSEA, DepMap proteomic (Wnt-high vs. Wnt-low)

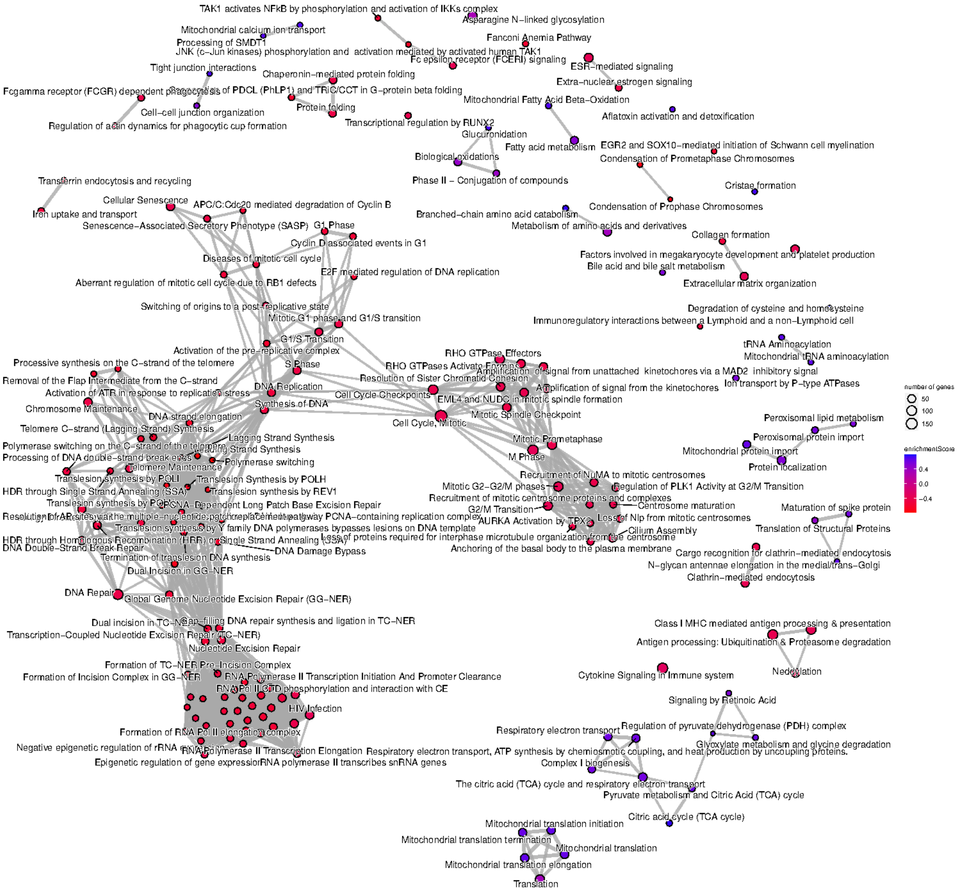

S5G) GO cellular component GSEA, DepMap transcriptomic (Wnt-high vs. Wnt-low)

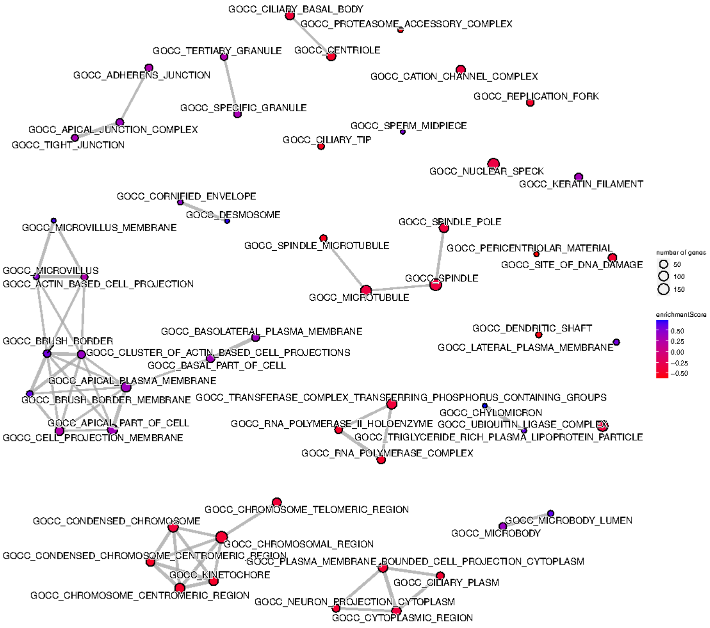

S5H) GO cellular component GSEA, DepMap proteomic (Wnt-high vs. Wnt-low)

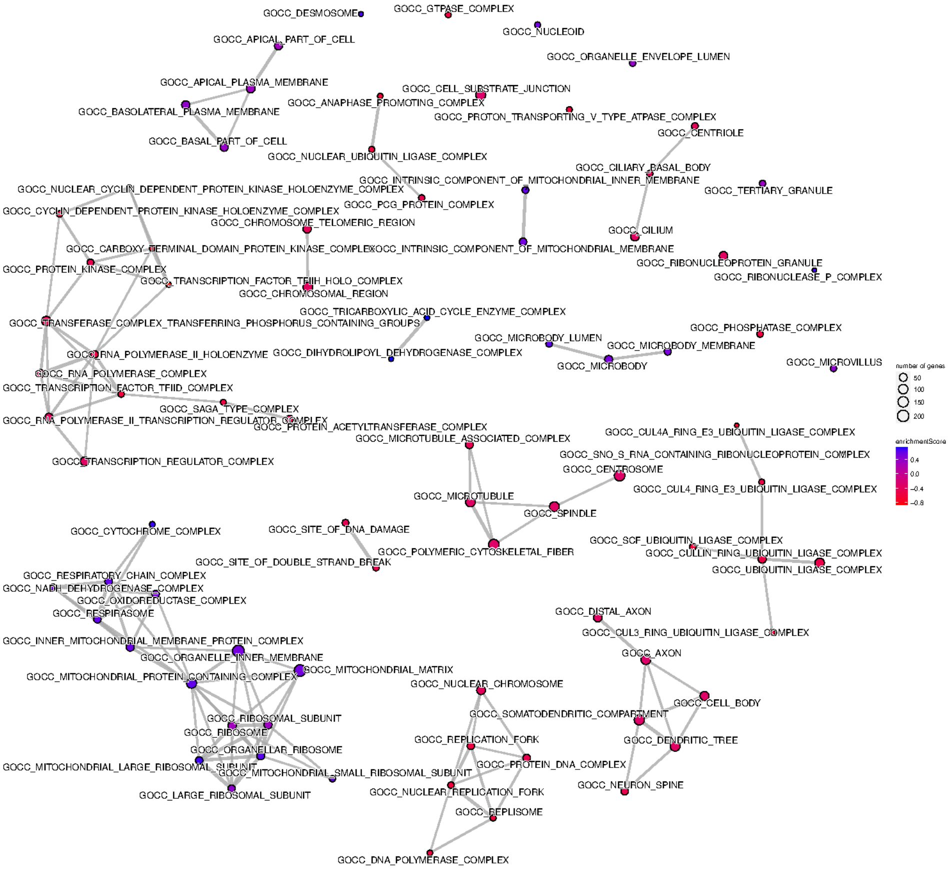

Appendix Figure S6

Transcript expression (A) and protein expression (B) of *SLC16A1* and *PDK1* (y-axis) compared to transcript expression of *AXIN2* (x-axis in the dotplot). Colors indicate CMS classification or normal colon tissue. Violin plots in the left panels illustrate candidate transcript and protein expression in the different CMS and tissue groups.

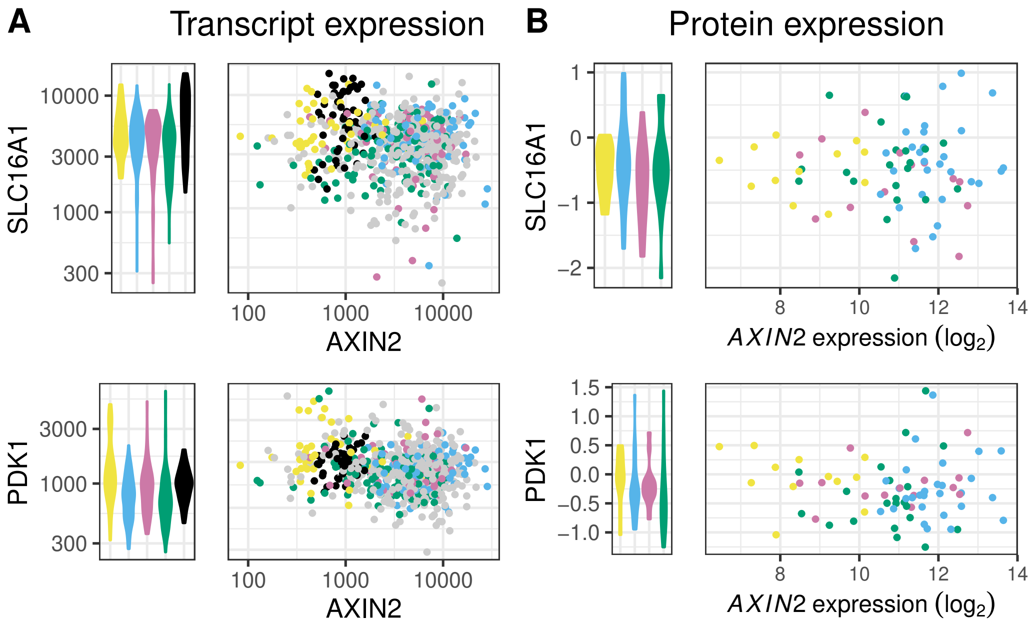

Supplement: Supplementary file 1 — Appendix [file MSB-18-e10874-s001.pdf]
