# Supplementary material for: Metabolic balance in colorectal cancer is maintained by optimal Wnt signaling levels
Source: Mol Syst Biol. 2022 Jul 29;18(8):e10874. doi: 10.15252/msb.202110874 (PMC9336172; doi:10.15252/msb.202110874)
Supplement: Supplementary file 2 [file MSB-18-e10874-s002.pdf]

molecular systems biology

# Metabolic balance in colorectal cancer is maintained by optimal Wnt signaling levels

Katharina Imkeller, Giulia Ambrosi, Nancy Klemm, Ainara Claveras Cabezudo, Luisa Henkel, Wolfgang Huber, and Michael Boutros
**DOI: 10.15252/msb.202110874**

*Corresponding author(s): Michael Boutros (m.boutros@dkfz.de) , Katharina Imkeller (imkeller@embl.de)*



## Review Timeline:

| | |
|---|---|
| Submission Date: | 15th Dec 21 |
| Editorial Decision: | 11th Feb 22 |
| Revision Received: | 14th May 22 |
| Editorial Decision: | 15th Jun 22 |
| Revision Received: | 3rd Jul 22 |
| Accepted: | 5th Jul 22 |

*Editor: Jingyi Hou*


## Transaction Report:

(Note: With the exception of the correction of typographical or spelling errors that could be a source of ambiguity, letters and reports are not edited. Depending on transfer agreements, referee reports obtained elsewhere may or may not be included in this compilation. Referee reports are anonymous unless the Referee chooses to sign their reports.)

Thank you for submitting your work to Molecular Systems Biology. We have now heard back from the three reviewers who agreed to evaluate your study. As you will see below, the reviewers find the topic of your study of potential interest. They raise, however, a series of concerns, which should be convincingly addressed in a major revision of the present manuscript.

I think the reviewers' recommendations are rather straightforward, and there is no need to reiterate all the points listed below. All issues raised by the reviewers need to be satisfactorily addressed. As you may already know, our editorial policy allows in principle a single round of major revision, and it is therefore essential to respond to the reviewers' comments that are as complete as possible. Please feel free to contact me in case you would like to discuss in further detail any of the issues raised by the reviewers.

On a more editorial level, we would ask you to address the following issues:

REFEREE REPORTS

--------------------------------------------------------

Reviewer #1:

In this study Imkeller et al performed whole-genome CRISPR_Cas9 screens with large-scale multiomic data to delineate functional subtypes of human colorectal cancer. They generated cell lines with APC loss-of-function mutations, resulting in hyperactivated Wnt signaling, and examined metabolic changes. Metabolic status in APC mutant cells with low endogenous Wnt

activity was different from the metabolic status of control cells which naturally acquired Wnt hyperactivation. They showed that the dosage level of oncogenic Wnt activity impacts the metabolic equilibrium and the mitochondrial phenotype of a cell type in a context-dependent manner.

Overall the experiments described herein are well controlled, and the conclusions are generally supported by the data. There are relatively minor issues that need to be addressed by the authors before publication.

Page 11:
"We found that Wnt-high colorectal cancer cell lines were more dependent on CTNNB1 and other members of the Wnt signaling pathway than Wnt-low colorectal cancer cell lines (Fig. 3B and C)." There are more than 20 Wnt-high and only 5 Wnt-low cell lines in the Fig3. C, therefore, the conclusion here cannot be inferred from what the plot shows. A statistical test is warranted here.

Page 12:
"Mean fold changes of negative control gRNAs (targeting luciferase) are displayed as black circles. Mean fold changes of negative control gRNAs (chromosome 10 promiscuous) are displayed as black triangles." Fig 3. F shows positive and negative controls but the figure legend describes both as negative controls. Also, chr10 promiscuous guides show high variability and are not a good positive control.

Fig4.
The plots are labeled incorrectly.

Page 15:
"Many of the genes involved in mitochondrial function had an important viability effect in all cell lines (Fig. 4D), which would have made it difficult to perform functional assays because of cell death. "
Functional assays for the genes involved in mitochondrial function with important viability effect could be done by collecting the cells at earlier timepoints. In this study they collected cells after 12 doubling times which is relatively a long time. To study essential genes in a cell, they could collect the cells after for example 8 doubling instead of 12.

Page 15:
"Knockout of LARS2, a gene encoding a mitochondrial tRNA synthetase, had a low to intermediate fitness effect in our cell lines (Fig. 4D) and was thus most suitable for use as a candidate gene for functional assays."
Here they are probably talking about plot 4E (instead of 4D), and they are other genes shown in the plot with low to intermediate fitness effect in the cell lines, but the authors did not clarify why LARS2 is the a uniquely suitable candidate gene. Indeed the *ARS2 mitochondrial tRNA synthetase genes would be expected to show similar phenotype, along with the mitochondrial ribosome.

Page 16:
"This result was reproducible using two different LARS2 targeting gRNAs (Fig. 5B)."
It would be better if they could show the mean of 2 gRNAs targeting LARS2 in replicate screens, instead of showing two different gRNAs targeting the gene. It does not show if they had the same result in different replicates.

Page 18:
"The tumors that were classified as CMS4 or CMS3 but belonged to the Wnt-low tumor subgroup showed metabolic gene and protein expression levels comparable to Wnt-low tumors classified as CMS1 (Fig. 6C and D)."
There is no figure 6C in the paper. Figure legends do not match panels.

Reviewer #2:

This study begins with an unbiased analysis of TCGA cohorts to stratify CMS according to Wnt signaling, which led to some discrepancies. However, stratification of the same cohorts into Wnt-low and Wnt-high was more informative. APC truncation was seen in Wnt-high CRC (80%), but less so in Wnt-low CRC (30%). Notably, Wnt-low CRC had high prevalence of other drivers, namely BRAF (40%) while RNF43 was also enriched in Wnt-low CRC. Stratification into Wnt-high and Wnt-low also revealed differences in MSI and tumor location. Importantly, the same stratification of CRC cell lines into Wnt-high and Wnt-low mirrored the cancers, indicating that the changes in gene expression were tumor cell intrinsic rather than due to the cellular components of the tumor microenvironment.
Intriguingly, engineered hyperactive Wnt signaling in Wnt-low cell lines (HCT116 and RKO) did not mirror natural hyperactive Wnt. Mutations in the beta-catenin gene and APC are mutually exclusive in CRC, thus introducing an APC truncation into HCT116 cells, which harbor mutations in the beta-catenin gene, does not mimic CRC. However, this model system revealed the complicity and context-dependence of Wnt signaling in CRC. The dependence on mitochondrial and metabolic states between

the natural and engineered Wnt hyperactivity is of particular importance as it enabled an unravelling of the underlying molecular mechanisms and revealed a reduced capacity to compensate for loss of mitochondrial function. The switch to Warburg occurs in both Wnt-high and Wnt-low tumors, but the degree of the shift differs between the two. Thus, the metabolic balance between glycolysis and oxidative respiration is intricately interwoven with the sequence of acquisition of mutations in CRC. Untangling this level of complexity was achieved in this study though comprehensive multi-omic analyses. This is an important, timely and enlightening study.

Minor comment:
Reference Fessler et al., 2016 in the last paragraph of the discussion is not referenced

Reviewer #3:

The authors present results from a hybrid experimental computational approach aiming at exploring the role of Wnt signalling dosage across colorectal cancer subtypes, and showing that hyper activated Wnt signalling leads to a different metabolic state than tumours with low Wnt activity, but also that engineered Wnt signaling leads to a metabolic state that is different than that led by naturally acquired Wnt hyper activation, implying context specificity and dependencies from the initial baseline metabolic state.

As a first step the authors identify two groups of tumours respectively with high/low levels of Wnt signalling and distinct mutational, molecular and transcriptional features by using public available data from the TCGA, and considering the basal expression levels of a transcriptional signatures of Wnt target genes.

Then the authors classify a panel of cell lines, using their publica available genomic/transcriptomic characterisation also in Wnt high/low groups, observing similar but less distinctive molecular features than those identified in the primary tumours associated with high Wnt levels.

The authors then go further, selecting two cell lines with low Wnt activation and putatively functional APC. They engineer these cell lines to hyper activate Wnt signaling via CRISPR mediated genetic disruption of APC.

Then, by differential expression analysis the authors find that the transcriptional changes brought about by engineered APC inactivation in these models are quite distinct from those obtained while contrasting endougenously Wnt-high cell lines versus Wnt-low cell lines. From this the authors conclude that the transcriptional footprints of Wnt activity are context dependant.

Going further, the authors seek to identify and compare genetic dependencies that are specific to their engineered Wnt-hyperactivated cell lines (when contrasted to parental cell lines) and those that are specific to endogenously Wnt-high cell lines (when compared to endogenously Wnt-low cell lines). To the first aim they perform a pooled genome wide CRISPR-cas9 screen on their APC-inactivated cell lines (APCtrunc cell lines) as well as on their parental APCwt cell lines and they contrast the obtained essentiality profiles. For the second aim the authors mined and contrasted public available essentiality profiles from the DepMap.

The authors have then further contrasted the differential essentiality profiles resulting from these two analyses and spot differences between Wnt-hyperactivation specific dependencies and endogenous Wnt-high activity dependencies. Finally the authors functionally characterise the dependencies that are specific to their engineered Wnt-hyperactivated models, reporting a synthetic lethality between APC truncations and mithocondrial genes.

Briefly, while the aim of this work is interesting and timely, as it detailed below, the presented pipeline and related results looks quite biased, guided by seemingly arbitrary choices prone to cherry picking and not considering several confounding factors.

In addition, in my opinion this work doesn't show that metabolic balance in colorectal cancer is maintained by optimal Wnt signaling levels but rather (and only) that artificially introduced oncogenic variants in APC lead to different downstreaming transcriptional changes than those triggered by APC variants endogenously present in cancer models, which by the way might have had the time to further optimise, by positive selection, their fitness via tuning the expression of other genes. This is a pretty obvious finding to me.

Furthermore the differential dependency analysis is performed in a quite sloppy way, comparing outcomes of screens performed with different sgRNA libraries (TKO vs AVANA, which of course have different and heterogenous sgRNA on/off-target efficiency), at different assay lengths (12 days vs 21 days) and with many other potential underlying differences in the experimental setting. All these are totally neglected by the authors but they can potentially lead to discrepancies (as shown in PMID: 31862961). Finally, particularly suspicious are to me the findings related to the specific dependencies in mitochondrial genes, whose essentiality has been shown to be particularly sensitive to the CRISPR screening length (PMID: 27760321)

Other (more specific) major points:

* The authors discriminate between Wnt low and high tumours via unsupervised clustering of transcriptional data of typical CTNNB1 dependent targets of Wnt following the observation that CMS classification is not sufficient to predict Wnt activation just by looking at the expression of a single gene (AXIN2). Shouldn't this claim be supported by showing plots similar to figure 1B for other Wnt targets? potentially as supplementary figures? In addition, I would quantify in someway the differential expression of the Wnt targets across CMS subtypes, to show this lack of predictive power and/or highlight (and quantify) the presence of Wnt active outliers in CMS3 and CMS4.

* In general an indication of the extent to which Wnt signalling led transcriptional differences participate to overall transcript CMS clusters is missing and would be nice to have.

* moreover how the optimal number of clusters (2) was determined? This is not specified, looks a priori determined, and by looking at the heat map in figure 1A, 3 clusters might have worked well too, with a third group (i.e. leftmost columns) perhaps composed by samples with intermediate levels of Wnt signaling or more ambiguous transcriptomic profiles of the the Wnt targets?

* Similarly, is not clear why the authors classified cell lines into Wnt low/high activity group based on the expression of one gene only instead of using unsupervised clustering of CTNNB1 dependent targets of Wnt, as they did for the primary tumours. This inconsistency looks suspicious and should be motivated.

* A much better/rigorous and state-of-the-art classification of cell lines into high/low Wnt group would have been an integrative cluster analysis of tumours/cell-lines together, to check also at what extent and which cell line mirror the two primary tumour groups. This could have been done for example following a Celligner normalisation, to remove batch effects and alling cell lines and primary tumours (as in PMID: 33397959). Alternatively signatures of differentially expressed genes could have been derived by contrasting low vs. high Wnt activity primary tumours and used as template classifiers for cell lines (as dane for example in PMID: 23584089). Using just AXIN2, looks overly simplistic and prone to cherry picking. The author should consider reperforming their cell line classification or alternatively they should provide a convincing argument against it.

* As a consequence, even the differential expression analyses results presented in figure 2B (and related comparison with those reported in figure 2I) could have been derived in a more rigorous way. In addition, the groups contrasted to produce figure 2B are never specified.

* The mutation prevalences shown in figures 1F to 1J look statistically significant, but this should be quantified by fisher/hypergeometric test. In addition, it is not clear if these associations were identified unbiasedly and systematically or in a prior knowledge guided way, just by focusing on selected mutations. This should be specified. Significance quantification would also allow comparing these results with those reported in figure2C to G.

* Terms like 'more ambiguous' (referring to CMS classification of cell lines vs tumours) and 'less pronounced' (referring to pattern of mutations of Wnt low/high cell lines) should be accompanied by statistical scores.

**MSB-2021-10874**
**Response to Reviewers**

We are glad to see that the three reviewers carefully assessed our manuscript and found our study interesting. Our point-by-point reply addresses the important issues that were raised and described the changes that have been incorporated into our revised manuscript.

**Reviewer #1:**

In this study Imkeller et al performed whole-genome CRISPR_Cas9 screens with large-scale multiomic data to delineate functional subtypes of human colorectal cancer. They generated cell lines with APC loss-of-function mutations, resulting in hyperactivated Wnt signaling, and examined metabolic changes. Metabolic status in APC mutant cells with low endogenous Wnt activity was different from the metabolic status of control cells which naturally acquired Wnt hyperactivation. They showed that the dosage level of oncogenic Wnt activity impacts the metabolic equilibrium and the mitochondrial phenotype of a cell type in a context-dependent manner.

Overall the experiments described herein are well controlled, and the conclusions are generally supported by the data. There are relatively minor issues that need to be addressed by the authors before publication.

*We thank the reviewer for the supportive comment.*

Page 11:
"We found that Wnt-high colorectal cancer cell lines were more dependent on CTNNB1 and other members of the Wnt signaling pathway than Wnt-low colorectal cancer cell lines (Fig. 3B and C)." There are more than 20 Wnt-high and only 5 Wnt-low cell lines in the Fig3. C, therefore, the conclusion here cannot be inferred from what the plot shows. A statistical test is warranted here.

*In the initial submission we performed a statistical test to show that the genes of the WNT_SIGNALING gene set are differentially essential between Wnt groups (Fig. 3B). Indeed, as pointed out by the reviewer, we did not specifically test the essentiality of CTNNB1, which is part of this gene set. We now performed a Wilcoxon-rank sum test (Fig. 3C) that shows that Wnt-high colorectal cancer cell lines are more dependent on CTNNB1 than Wnt-low colorectal cancer cell lines (described in Figure legend).*

*OF NOTE: In the revised version of our manuscript use a more recent version of the DepMap dataset (21Q3), which means that we were able to include more cell lines into the analysis. The analysis scripts that were part of the initial submission remained unchanged.*

Page 12:
"Mean fold changes of negative control gRNAs (targeting luciferase) are displayed as black circles. Mean fold changes of negative control gRNAs (chromosome 10 promiscuous) are displayed as black triangles." Fig 3. F shows positive and negative controls but the figure

legend describes both as negative controls. Also, chr10 promiscuous guides show high variability and are not a good positive control.

*We agree with the reviewer. The "chromosome 10 promiscuous" gRNAs were called "negative control" by mistake. We adapted the Figure legend of Figure 3F and 4D accordingly and scanned the manuscript to make sure that this error did not occur anywhere else.*

Fig4.
The plots are labeled incorrectly.

*We apologize for the mistake we made and have now relabeled the figure elements accordingly.*

Page 15:
"Many of the genes involved in mitochondrial function had an important viability effect in all cell lines (Fig. 4D), which would have made it difficult to perform functional assays because of cell death. " Functional assays for the genes involved in mitochondrial function with important viability effect could be done by collecting the cells at earlier timepoints. In this study they collected cells after 12 doubling times which is relatively a long time. To study essential genes in a cell, they could collect the cells after for example 8 doubling instead of 12.

*The 12 doubling times mentioned by the reviewer apply to the whole genome CRISPR screen, which was started 3 days after viral transduction with the gRNA library. All functional assays in our work were started 3-5 days (i.e. maximum ca. 5 doublings) after viral transduction. For the competitive growth assay, the cell pools were collected and analysed 2, 5, 7, 9, 12, 14 and 17 days after transduction. The Seahorse measurement of respiration was started 5 days after transduction. The procedure suggested by the reviewer was thus already in place, when we performed the experiments.*

*In order to avoid confusion and also to clarify the rationale behind the candidate gene selection, we have reformulated the text, also with respect to the next comment of the reviewer. The sentence "Many of the genes involved in mitochondrial function had an important viability effect in all cell lines (Fig. 4D), which would have made it difficult to perform functional assays because of cell death." was removed and replaced by an improved explanation for the candidate gene selection (Page 15).*

Page 15:
"Knockout of LARS2, a gene encoding a mitochondrial tRNA synthetase, had a low to intermediate fitness effect in our cell lines (Fig. 4D) and was thus most suitable for use as a candidate gene for functional assays." Here they are probably talking about plot 4E (instead of 4D), and they are other genes shown in the plot with low to intermediate fitness effect in the cell lines, but the authors did not clarify why LARS2 is the a uniquely suitable candidate gene. Indeed the *ARS2 mitochondrial tRNA synthetase genes would be expected to show similar phenotype, along with the mitochondrial ribosome.

*As indicated above, and as pointed out by the reviewer, we have relabeled the panels in Figure 4. Figure 4B and 4C show a number of genes with intermediate differential fitness effects. The genes related to mitochondrial function are highlighted in black, since we observed in the gene set enrichment analysis, that there was an effect for pathways affecting mitochondria. Selected genes are highlighted and labeled in green in all figures 4B,C,E,F, to allow the reader to see reproducible effects between both analyses.*

*We chose LARS2 as one example candidate gene involved in mitochondrial function but we agree with the reviewer: other genes involved in mitochondrial tRNA synthesis are expected to show the same phenotype. We reformulated the text accordingly to clarify that LARS2 is not the most suitable candidate, but rather one of many suitable candidates (Page 15).*

Page 16:
"This result was reproducible using two different LARS2 targeting gRNAs (Fig. 5B)."
It would be better if they could show the mean of 2 gRNAs targeting LARS2 in replicate screens, instead of showing two different gRNAs targeting the gene. It does not show if they had the same result in different replicates.

*Fig. 5B summarizes the results of three independent replicates of the competitive growth assay illustrated in Fig. 5A. The assay is performed separately for the two gRNAs and cell lines and with independent virus transduction for each replicate. Displaying the result as the mean of the effect of the two gRNAs does not make sense here, because the gRNAs were measured in different assays. In fact, we think that displaying the results by gRNA as done in present Fig. 5B emphasizes how reproducible the observation is in different experiments and with different gRNAs. The reproducibility between replicates can be judged from the data points in Fig. 5B, where each point represents one independent replicate. We have included an explanatory statement in the figure legend and we hope that the reviewer agrees with our choice after this clarification.*

Page 18:
"The tumors that were classified as CMS4 or CMS3 but belonged to the Wnt-low tumor subgroup showed metabolic gene and protein expression levels comparable to Wnt-low tumors classified as CMS1 (Fig. 6C and D)." There is no figure 6C in the paper. Figure legends do not match panels.

*We apologize for the mistake we made and have now relabeled the figure elements accordingly.*

**Reviewer #2:**

This study begins with an unbiased analysis of TCGA cohorts to stratify CMS according to Wnt signaling, which led to some discrepancies. However, stratification of the same cohorts into Wnt-low and Wnt-high was more informative. APC truncation was seen in Wnt-high CRC (80%), but less so in Wnt-low CRC (30%). Notably, Wnt-low CRC had high prevalence of other drivers, namely BRAF (40%) while RNF43 was also enriched in Wnt-low CRC.

Stratification into Wnt-high and Wnt-low also revealed differences in MSI and tumor location. Importantly, the same stratification of CRC cell lines into Wnt-high and Wnt-low mirrored the cancers, indicating that the changes in gene expression were tumor cell intrinsic rather than due to the cellular components of the tumor microenvironment.
Intriguingly, engineered hyperactive Wnt signaling in Wnt-low cell lines (HCT116 and RKO) did not mirror natural hyperactive Wnt. Mutations in the beta-catenin gene and APC are mutually exclusive in CRC, thus introducing an APC truncation into HCT116 cells, which harbor mutations in the beta-catenin gene, does not mimic CRC. However, this model system revealed the complicity and context-dependence of Wnt signaling in CRC. The dependence on mitochondrial and metabolic states between the natural and engineered Wnt hyperactivity is of particular importance as it enabled an unravelling of the underlying molecular mechanisms and revealed a reduced capacity to compensate for loss of mitochondrial function. The switch to Warburg occurs in both Wnt-high and Wnt-low tumors, but the degree of the shift differs between the two. Thus, the metabolic balance between glycolysis and oxidative respiration is intricately interwoven with the sequence of acquisition of mutations in CRC. Untangling this level of complexity was achieved in this study though comprehensive multi-omic analyses. This is an important, timely and enlightening study.

*We thank the reviewer for the supporting comments.*

Minor comment:
Reference Fessler et al., 2016 in the last paragraph of the discussion is not referenced

*We thank the reviewer for pointing this out. The reference regarding differences between Wnt signaling induction in intestinal stem cells compared to more differentiated cells is now included in the text as well as in the list of references.*

**Reviewer #3:**

The authors present results from a hybrid experimental computational approach aiming at exploring the role of Wnt signalling dosage across colorectal cancer subtypes, and showing that hyper activated Wnt signalling leads to a different metabolic state than tumours with low Wnt activity, but also that engineered Wnt signaling leads to a metabolic state that is different than that led by naturally acquired Wnt hyper activation, implying context specificity and dependencies from the initial baseline metabolic state.

As a first step the authors identify two groups of tumours respectively with high/low levels of Wnt signalling and distinct mutational, molecular and transcriptional features by using public available data from the TCGA, and considering the basal expression levels of a transcriptional signatures of Wnt target genes.

Then the authors classify a panel of cell lines, using their publica available genomic/transcriptomic characterisation also in Wnt high/low groups, observing similar but less distinctive molecular features than those identified in the primary tumours associated with high Wnt levels.

The authors then go further, selecting two cell lines with low Wnt activation and putatively functional APC. They engineer these cell lines to hyper activate Wnt signaling via CRISPR mediated genetic disruption of APC.

Then, by differential expression analysis the authors find that the transcriptional changes brought about by engineered APC inactivation in these models are quite distinct from those obtained while contrasting endougenously Wnt-high cell lines versus Wnt-low cell lines. From this the authors conclude that the transcriptional footprints of Wnt activity are context dependant.

Going further, the authors seek to identify and compare genetic dependencies that are specific to their engineered Wnt-hyperactivated cell lines (when contrasted to parental cell lines) and those that are specific to endogenously Wnt-high cell lines (when compared to endogenously Wnt-low cell lines). To the first aim they perform a pooled genome wide CRISPR-cas9 screen on their APC-inactivated cell lines (APCtrunc cell lines) as well as on their parental APCwt cell lines and they contrast the obtained essentiality profiles. For the second aim the authors mined and contrasted public available essentiality profiles from the DepMap.

The authors have then further contrasted the differential essentiality profiles resulting from these two analyses and spot differences between Wnt-hyperactivation specific dependencies and endogenous Wnt-high activity dependencies. Finally the authors functionally characterise the dependencies that are specific to their engineered Wnt-hyperactivated models, reporting a synthetic lethality between APC truncations and mithocondrial genes.

Briefly, while the aim of this work is interesting and timely, as it detailed below, the presented pipeline and related results looks quite biased, guided by seemingly arbitrary choices prone to cherry picking and not considering several confounding factors.

In addition, in my opinion this work doesn't show that metabolic balance in colorectal cancer is maintained by optimal Wnt signaling levels but rather (and only) that artificially introduced oncogenic variants in APC lead to different downstreaming transcriptional changes than those triggered by APC variants endogenously present in cancer models, which by the way might have had the time to further optimise, by positive selection, their fitness via tuning the expression of other genes. This is a pretty obvious finding to me.

*The reviewer here summarizes a very important aspect of our work, which is the context dependency of Wnt signaling effects. However, we would like to point out that the reviewer's comment unfortunately only covers and discusses an incomplete part of our work and findings.*

*When referring to the transcriptional and functional genomic difference between engineered and endogenous effects of APC variants, the reviewer correctly summarizes one of our main findings. Context dependency results in different downstream effects depending on whether an APC truncation was acquired naturally during tumorigenesis or whether it was introduced artificially into tumor cells that underwent an alternative path of tumorigenesis. Although this finding in itself does not yet provide a biological explanation of tumorigenesis, it is a very critical finding that challenges the use of certain model systems to study Wnt signaling in colorectal cancer. This is an important and highly non-trivial result with immediate implications for the functional genomics and Wnt signaling communities.*

*In addition, our work goes much further than this and also provides evidence for a direct link between metabolic balance and Wnt signaling levels. In Figure 6 we show transcriptomic and proteomic differences in metabolic pathways between tumor tissues with different levels of Wnt signaling. In the experiment depicted in Fig. 5D, where we measure respiration shortly after ligand-mediated Wnt signaling activation, we clearly show that there is a direct link between Wnt signaling and mitochondrial function. Our findings thus clearly provide an explanation for why Wnt signaling levels need to be optimal in order to maintain the metabolic balance in cancer cells.*

Furthermore the differential dependency analysis is performed in a quite sloppy way, comparing outcomes of screens performed with different sgRNA libraries (TKO vs AVANA, which of course have different and heterogenous sgRNA on/off-target efficiency), at different assay lengths (12 days vs 21 days) and with many other potential underlying differences in the experimental setting. All these are totally neglected by the authors but they can potentially lead to discrepancies (as shown in PMID: 31862961). Finally, particularly suspicious are to me the findings related to the specific dependencies in mitochondrial genes, whose essentiality has been shown to be particularly sensitive to the CRISPR screening length (PMID: 27760321)

*We agree with the reviewer, that gRNA libraries and screening length affect hit detection in whole-genome CRISPR screens. However, the reviewer does not explain how this would affect the analyses and results that we obtain in our study. In fact we are aware of these well-known technical effects and thus are very careful*

*when comparing the results of our screen to other data. We keep all analyses and statistical models for our screen and the DepMap screen well apart.*

*Moreover, our own CRISPR screen is very well controlled, and we did everything possible to keep technical variability to a minimum. The screen duration in units of duplication time is exactly the same in all cell lines and the cell lines have very similar growth rates. It is thus excluded that differences in experimental settings are the reason why RKO-APCtrunc and HCT116-APCtrunc are more vulnerable to mitochondrial perturbation than their wildtype counterparts. In addition, our functional assays (competitive growth assay and respiration measurement, Fig. 5) unequivocally show that there is a difference in mitochondrial function between APC and WT cell lines.*

*Since this is a very critical point, we had already included a section in the discussion to argue why we are confident that our conclusions are not biased by technical artifacts: "The metabolic phenotype of Wnt-low tumors correlated with a higher vulnerability towards mitochondrial perturbation. Given the statistical model that we used to compare the gRNA abundances (Fig. S2) as well as the successful validation using the competitive cell growth assay (Fig. 5B), we are confident that this observation reflects the underlying biology and is not linked to technical artifacts that arise from differences in growth rate during the vulnerability screen." (Page 23)*

Other (more specific) major points:

* The authors discriminate between Wnt low and high tumours via unsupervised clustering of transcriptional data of typical CTNNB1 dependent targets of Wnt following the observation that CMS classification is not sufficient to predict Wnt activation just by looking at the expression of a single gene (AXIN2). Shouldn't this claim be supported by showing plots similar to figure 1B for other Wnt targets? potentially as supplementary figures? In addition, I would quantify in someway the differential expression of the Wnt targets across CMS subtypes, to show this lack of predictive power and/or highlight (and quantify) the presence of Wnt active outliers in CMS3 and CMS4.

*We thank the reviewer for this valuable suggestion. We now include a principal component analysis to illustrate the limited contribution of Wnt target gene expression to CMS subtype classification. We also include other visualizations similar to Figure 1B for other Wnt target genes. These analyses can be found in the newly introduced Figure S1.*

* In general an indication of the extent to which Wnt signalling led transcriptional differences participate to overall transcript CMS clusters is missing and would be nice to have.

*The CMS assignment of tumor tissue transcription profiles is based on a random forest model as implemented in the CMSclassifier package and as described in the methods section. The algorithm operates on a matrix of whole transcriptome expression values. It is not clear to us how the reviewer would like us to show the contribution of Wnt-target gene expression to this classification algorithm. The expression of Wnt target genes in the different samples and their classification into*

*CMS is illustrated in Fig. 1A. Also, the newly introduced figure S1 illustrates how Wnt target gene expression and CMS classification relate to each other.*

* moreover how the optimal number of clusters (2) was determined? This is not specified, looks a priori determined, and by looking at the heat map in figure 1A, 3 clusters might have worked well too, with a third group (i.e. leftmost columns) perhaps composed by samples with intermediate levels of Wnt signaling or more ambiguous transcriptomic profiles of the the Wnt targets?

*We did not determine the optimal number of clusters in Fig. 1A. We decided to classify the tumors and cell lines into Wnt-high and Wnt-low in order to make our analyses and conclusions easier to follow. In our manuscript we focus on functional genomics approaches to illustrate a complex interplay between signaling and metabolism in different model systems. The clustering of tumors and cell lines is not the main point of our work and there are certainly also other ways to perform clustering. Nevertheless our approach is good enough to conduct our downstream analyses. We validate our choice by quantifying the expected cancer driver mutations for Wnt-high and Wnt-low tumors (Fig. 1E-J). For the cell lines, we verify that Wnt-high cells are indeed more dependent on Wnt signaling than Wnt-low cells (Fig. 3B+C).*

* Similarly, is not clear why the authors classified cell lines into Wnt low/high activity group based on the expression of one gene only instead of using unsupervised clustering of CTNNB1 dependent targets of Wnt, as they did for the primary tumours. This inconsistency looks suspicious and should be motivated.

*The reply to this comment is combined with the reply to the following comment, because both comments address the same question, namely the classification of cell lines.*

* A much better/rigorous and state-of-the-art classification of cell lines into high/low Wnt group would have been an integrative cluster analysis of tumours/cell-lines together, to check also at what extent and which cell line mirror the two primary tumour groups. This could have been done for example following a Celligner normalisation, to remove batch effects and alling cell lines and primary tumours (as in PMID: 33397959). Alternatively signatures of differentially expressed genes could have been derived by contrasting low vs. high Wnt activity primary tumours and used as template classifiers for cell lines (as dane for example in PMID: 23584089). Using just AXIN2, looks overly simplistic and prone to cherry picking. The author should consider reperforming their cell line classification or alternatively they should provide a convincing argument against it.

*The classification of cell lines into Wnt high and Wnt low groups is meant to separate tumor cells that underwent Wnt-dependent tumorigenesis from those who underwent Wnt-independent tumorigenesis. As stated throughout the manuscript, we here focus on classical CTNNB1-dependent Wnt signaling. It is widely accepted that the canonical Wnt-dependent pathway of tumorigenesis is distinct from the serrated pathway, for example in terms of tumor localization, microsatellite instability and cancer driver mutations (for example reviewed in PMID 28741479). In Figures 1E-J*

*and 2C-G, we demonstrate that our classification method for tumors and cell lines is suitable to reproduce these patterns.*

*For classification of cell lines, we use AXIN2 expression levels since it has been shown that AXIN2 is the main target gene of CTNNB1-dependendent classical Wnt signaling. It is very consistently upregulated upon classical Wnt activation in multiple different cell types and tissue and is therefore not as prone to context-dependent effects as the other members of the Wnt target gene set. Moreover, clustering on expression of Wnt-target gene expression in cell lines does not work very well because some of the Wnt target genes are not expressed in cell lines, or only expressed at a very low level. This might be due to the fact that the tissue hierarchy is important for paracrine and juxtacrine Wnt signaling. We believe that AXIN2 expression is a valid criterion for quantifying classical Wnt signaling, also because our grouping also shows a higher dependence on CTNNB1 in Wnt high cell lines compared to Wnt low (Fig. 3C). Taken together all of this, we believe that our classification method is sufficient to distinguish between both tumor and cell line groups and we do not see an urgent need for applying another classification method.*

*In fact, even though the methods suggested by the reviewer integrate cell line with tumor expression data and are very useful and valuable for studying cancer signaling, we believe that they do not constitute the right approach in particular to study Wnt signaling. Paracrine and juxtacrine mediate and maintain Wnt signaling networks in tumor tissue, which leads to Wnt target gene expression patterns that cannot be observed in cell cultures that most likely lack structured tissue signaling hierarchy. We therefore believe that from a biological point of view, it would not make sense to choose a method that aligns expression profiles from highly organized tissues with those of cell line cultures to study Wnt target gene expression. Indeed, we observed in the analysis results provided by Celliner, that the combined analysis of tumor and cell lines and subsequent dimension reduction and/or clustering does not distinguish between Wnt dependent and Wnt independent pathway of tumorigenesis. Also, when using the Celliner function to find the most similar tumor for a set of colorectal cancer cell lines, we frequently found low distances reported for unrelated tumor lineages, especially for the cell lines without APC truncation (for example RKO - uterus or HCT116 - gastric).*

*In summary, we believe that given our results on CTNNB1 dependency, our cell line classification approach is suitable for our analyses and is well supported from a biological point of view. In our study, where we describe the context dependent effects of a pathway in which tissue architecture and paracrine signaling play an important role, we do not see an advantage from using approaches that align tumor and cell line transcriptomes. The obvious limitations of cell line model systems in comparison to actual tumor tissue cannot be overcome by data integration.*

* As a consequence, even the differential expression analyses results presented in figure 2B (and related comparison with those reported in figure 2I) could have been derived in a more rigorous way. In addition, the groups contrasted to produce figure 2B are never specified.

*Figure 2B depicts the "differential gene expression analysis comparing Wnt-high (n = 8 cell lines) versus Wnt-low (n = 39 cell lines) CRC cell lines". These groups are defined in Figure 2A.*

* The mutation prevalences shown in figures 1F to 1J look statistically significant, but this should be quantified by fisher/hypergeometric test. In addition, it is not clear if these associations were identified unbiasedly and systematically or in a prior knowledge guided way, just by focusing on selected mutations. This should be specified. Significance quantification would also allow comparing these results with those reported in figure2C to G.

*The selection of mutations depicted here has been done in a prior-knowledge guided way, based on genetic alterations linked to different CRC subtypes (cite). We have modified the text accordingly: "Different frequencies of well-known CRC-driver mutation could be observed". We have included statistical tests for Figures 1F-J and 2C-G.*

* Terms like 'more ambiguous' (referring to CMS classification of cell lines vs tumours) and 'less pronounced' (referring to pattern of mutations of Wnt low/high cell lines) should be accompanied by statistical scores.

*We have replaced the term "more ambiguous" with "more difficult" and added an explanation why the classification is more difficult for cell lines: "presumably due to lack of immune and stromal infiltration in cell lines, which contribute to a certain degree to the transcriptional signature of CMS in tumors".*

*We have removed the expression "less pronounced" for the mutation patterns. Since we included the statistical tests for Figure 1F and 1J, we think that the modified sentence "The patterns of cancer driver mutations in Wnt-low and Wnt-high colorectal cancer cell lines were partially comparable to the ones observed in the tumor tissue." is sufficient. We reformulated the paragraph describing the differences accordingly.*

Dear Michael,

Thank you for sending us your revised manuscript. We have now heard back from the two reviewers who agreed to evaluate your study. As you will see, the reviewers are overall satisfied with the modifications made.

Before we can formally accept your manuscript, we would ask you to address the following issues:

REFEREE REPORTS
---------------------------------------------------------------------------

Reviewer #1:

Authors have responded sufficiently to my concerns, and appear to have robustly addressed criticisms from other reviewers.

Reviewer #3:

The authors have sufficiently addressed most of my points and clarified some aspects of their analysis in their point-by-point responses to reviewers comments.

In my previous review I expressed a major criticism about this work not showing that metabolic balance in colorectal cancer is maintained by optimal Wnt signaling levels but rather (and only) that artificially introduced oncogenic variants in APC lead to different downstreaming transcriptional changes than those triggered by APC variants endogenously present in cancer models, and that these might have had the time to further optimise, by positive selection, their fitness via tuning the expression of other genes.

The authors agree with this claim but they also replied that "although this finding in itself does not yet provide a biological explanation of tumorigenesis, it is a very critical finding that challenges the use of certain model systems to study Wnt signaling in colorectal cancer."
I do believe that seen under this prospective, while still valid and of potential impact, the take-home message of this manuscript should be re-elaborated and properly re-discussed, potentially even changing the title of the the manuscript.

**RESPONSE TO THE REVIEWERS**

Reviewer #1:

Authors have responded sufficiently to my concerns, and appear to have robustly addressed criticisms from other reviewers.

*We thank the reviewer for the positive feedback.*

Reviewer #3:

The authors have sufficiently addressed most of my points and clarified some aspects of their analysis in their point-by-point responses to reviewers comments.

In my previous review I expressed a major criticism about this work not showing that metabolic balance in colorectal cancer is maintained by optimal Wnt signaling levels but rather (and only) that artificially introduced oncogenic variants in APC lead to different downstreaming transcriptional changes than those triggered by APC variants endogenously present in cancer models, and that these might have had the time to further optimise, by positive selection, their fitness via tuning the expression of other genes.

The authors agree with this claim but they also replied that "although this finding in itself does not yet provide a biological explanation of tumorigenesis, it is a very critical finding that challenges the use of certain model systems to study Wnt signaling in colorectal cancer."

I do believe that seen under this prospective, while still valid and of potential impact, the take-home message of this manuscript should be re-elaborated and properly re-discussed, potentially even changing the title of the the manuscript.

*We agree with Reviewer 3 on the fact that our manuscript shows that different downstream effects are observed, depending on whether an APC truncation is acquired naturally during tumorigenesis or whether it is introduced artificially into tumor cells.*

*As far as we understand, Reviewer 3 would like to see that we also illustrate the interplay between Wnt signaling and metabolism. Indeed, our work provides evidence for this direct link between mitochondrial function and Wnt signaling levels (Figures 6 and 5D). "In Figure 6 we show transcriptomic and proteomic differences in metabolic pathways between tumor tissues with different levels of Wnt signaling. In the experiment depicted in Fig. 5D, where we measure respiration shortly after ligand-mediated Wnt signaling activation, we clearly show that there is a direct link between Wnt signaling and mitochondrial function." (cited from our 1st response to the reviewers)*

*Finally, we would like to mention that the fact that cells "optimise, by positive selection, their fitness via tuning the expression of other genes", as suggested by the reviewer, is totally in the scope of the work presented and discussed in our manuscript. The mechanism, by which Wnt signaling and metabolic balance are*

*linked, is not addressed in the manuscript. But certainly - and here we agree with the reviewer - positive selection and transcriptional regulation may play a role in it.*

**ACCEPTED** **5th Jul 2022**

Thank you again for sending us your revised manuscript. We are now satisfied with the modifications made and I am pleased to inform you that your paper has been accepted for publication.

EMBO PRESS

YOU MUST COMPLETE ALL CELLS WITH A PINK BACKGROUND 🡻

PLEASE NOTE THAT THIS CHECKLIST WILL BE PUBLISHED ALONGSIDE YOUR PAPER

| |
|---|
| Corresponding Author Name: Michael Boutros |
| Journal Submitted to: Molecular Systems Biology |
| Manuscript Number: MSB-2021-10874 |

## Reporting Checklist For Life Sciences Articles (Rev. June 2017)

This checklist is used to ensure good reporting standards and to improve the reproducibility of published results. These guidelines are consistent with the Principles and Guidelines for Reporting Preclinical Research issued by the NIH in 2014. Please follow the journal's authorship guidelines in preparing your manuscript.

### A- Figures
### 1. Data
### The data shown in figures should satisfy the following conditions:

- the data were obtained and processed according to the field's best practice and are presented to reflect the results of the experiments in an accurate and unbiased manner.
- figure panels include only data points, measurements or observations that can be compared to each other in a scientifically meaningful way.
- graphs include clearly labeled error bars for independent experiments and sample sizes. Unless justified, error bars should not be shown for technical replicates.
- if n< 5, the individual data points from each experiment should be plotted and any statistical test employed should be justified
- Source Data should be included to report the data underlying graphs. Please follow the guidelines set out in the author ship guidelines on Data Presentation.

### 2. Captions

### Each figure caption should contain the following information, for each panel where they are relevant:

- a specification of the experimental system investigated (eg cell line, species name).
- the assay(s) and method(s) used to carry out the reported observations and measurements
- an explicit mention of the biological and chemical entity(ies) that are being measured.
- an explicit mention of the biological and chemical entity(ies) that are altered/varied/perturbed in a controlled manner.
- the exact sample size (n) for each experimental group/condition, given as a number, not a range;
- a description of the sample collection allowing the reader to understand whether the samples represent technical or biological replicates (including how many animals, litters, cultures, etc.).
- a statement of how many times the experiment shown was independently replicated in the laboratory.
- definitions of statistical methods and measures:
  - common tests, such as t-test (please specify whether paired vs. unpaired), simple χ2 tests, Wilcoxon and Mann-Whitney tests, can be unambiguously identified by name only, but more complex techniques should be described in the methods section;
  - are tests one-sided or two-sided?
  - are there adjustments for multiple comparisons?
  - exact statistical test results, e.g., P values = x but not P values < x;
  - definition of 'center values' as median or average;
  - definition of error bars as s.d. or s.e.m.

Any descriptions too long for the figure legend should be included in the methods section and/or with the source data.

**In the pink boxes below, please ensure that the answers to the following questions are reported in the manuscript itself. Every question should be answered. If the question is not relevant to your research, please write NA (non applicable). We encourage you to include a specific subsection in the methods section for statistics, reagents, animal models and human subjects.**

## USEFUL LINKS FOR COMPLETING THIS FORM

| | |
|---|---|
| http://www.antibodypedia.com | Antibodypedia |
| http://1degreebio.org | 1DegreeBio |
| http://www.equator-network.org/reporting-guidelines/improving-bioscience-research-reporti | ARRIVE Guidelines |
| http://grants.nih.gov/grants/olaw/olaw.htm | NIH Guidelines in animal use |
| http://www.mrc.ac.uk/Ourresearch/Ethicsresearchguidance/Useofanimals/index.htm | MRC Guidelines on animal use |
| http://ClinicalTrials.gov | Clinical Trial registration |
| http://www.consort-statement.org | CONSORT Flow Diagram |
| http://www.consort-statement.org/checklists/view/32-consort/66-title | CONSORT Check List |
| http://www.equator-network.org/reporting-guidelines/reporting-recommendations-for-tumor | REMARK Reporting Guidelines (marker prognostic studies) |
| http://datadryad.org | Dryad |
| http://figshare.com | Figshare |
| http://www.ncbi.nlm.nih.gov/gap | dbGAP |
| http://www.ebi.ac.uk/ega | EGA |
| http://biomodels.net/ | Biomodels Database |
| http://biomodels.net/miriam/ | MIRIAM Guidelines |
| http://jjj.biochem.sun.ac.za | JWS Online |
| https://osp.od.nih.gov/biosafety-biosecurity-and-emerging-biotechnology/ | Biosecurity Documents from NIH |
| http://www.selectagents.gov/ | List of Select Agents |

### B- Statistics and general methods

| | Please fill out these boxes 🡻 (Do not worry if you cannot see all your text once you press return) |
|---|---|
| 1.a. How was the sample size chosen to ensure adequate power to detect a pre-specified effect size? | We did not perform sample-size calculations. Public datasets used in this work include > 10 different samples. The CRISPR screen was performed in two independent replicates with multiple gRNAs per gene. We perform multiple-testing correction where applicable. |
| 1.b. For animal studies, include a statement about sample size estimate even if no statistical methods were used. | Our work does not include animal studies. |
| 2. Describe inclusion/exclusion criteria if samples or animals were excluded from the analysis. Were the criteria pre-established? | No samples or data were excluded from the analysis in general. In some cases (indicated in the manuscript when applicable), we restricted the analysis on sampkes that were sequenced at a given read length, in order to avoid batch effects from differences in sequencing approach. |
| 3. Were any steps taken to minimize the effects of subjective bias when allocating animals/samples to treatment (e.g. randomization procedure)? If yes, please describe. | The experimental procedures used in the work are in general such that subjective bias is unlikely. Randomization of sample localization was performed in experiments involving plates in order to avoid technical biases as much as possible. |
| For animal studies, include a statement about randomization even if no randomization was used. | Our work does not include animal studies. |
| 4.a. Were any steps taken to minimize the effects of subjective bias during group allocation or/and when assessing results (e.g. blinding of the investigator)? If yes please describe. | No blinding was performed. The data acquisition and analysis was sufficiently automatized to avoid related biases. |
| 4.b. For animal studies, include a statement about blinding even if no blinding was done | Our work does not include animal studies. |
| 5. For every figure, are statistical tests justified as appropriate? | Yes. |
| Do the data meet the assumptions of the tests (e.g., normal distribution)? Describe any methods used to assess it. | The data presented in Figure 5 meets the assumptions of statistical test used. The statistical models used for differential gene expression analysis and gene set enrichment analysis were designed for the specific data type and application, and thus no general assumptions need to be tested. |
| Is there an estimate of variation within each group of data? | The variation of data presented in Figure 5 are indicated in the box plots. The variance for other data types was estimated as part of the statistical models used in the analysis. |
| Is the variance similar between the groups that are being statistically compared? | Yes. |

### C- Reagents

| | |
|---|---|
| 6. To show that antibodies were profiled for use in the system under study (assay and species), provide a citation, catalog number and/or clone number, supplementary information or reference to an antibody validation profile. e.g., Antibodypedia (see link list at top right), 1DegreeBio (see link list at top right). | 1. Monoclonal mouse anti-human-APC antibody: Ali12-28, sc-53165, Lot#A2814, Santa Cruz Biotechnology, raised against amino acids 1-433 of human APC.<br>2. Goat anti-mouse IgG HRP, AB_10015289, Jackson ImmunoResearch. |
| 7. Identify the source of cell lines and report if they were recently authenticated (e.g., by STR profiling) and tested for mycoplasma contamination. | HCT116 and RKO cells were obtained from ATCC. Authentication of genotypes was performed by SNP profiling (Multiplexion, Heidelberg). Absence of mycoplasma infection was confirmed by regular testing. |

* for all hyperlinks, please see the table at the top right of the document

## D- Animal Models

| | |
|---|---|
| 8. Report species, strain, gender, age of animals and genetic modification status where applicable. Please detail housing and husbandry conditions and the source of animals. | Our work does not include animal studies. |
| 9. For experiments involving live vertebrates, include a statement of compliance with ethical regulations and identify the committee(s) approving the experiments. | - |
| 10. We recommend consulting the ARRIVE guidelines (see link list at top right) (PLoS Biol. 8(6), e1000412, 2010) to ensure that other relevant aspects of animal studies are adequately reported. See author guidelines, under 'Reporting Guidelines'. See also: NIH (see link list at top right) and MRC (see link list at top right) recommendations. Please confirm compliance. | - |

## E- Human Subjects

| | |
|---|---|
| 11. Identify the committee(s) approving the study protocol. | Our work does not include primary human subject studies. |
| 12. Include a statement confirming that informed consent was obtained from all subjects and that the experiments conformed to the principles set out in the WMA Declaration of Helsinki and the Department of Health and Human Services Belmont Report. | - |
| 13. For publication of patient photos, include a statement confirming that consent to publish was obtained. | - |
| 14. Report any restrictions on the availability (and/or on the use) of human data or samples. | - |
| 15. Report the clinical trial registration number (at ClinicalTrials.gov or equivalent), where applicable. | - |
| 16. For phase II and III randomized controlled trials, please refer to the CONSORT flow diagram (see link list at top right) and submit the CONSORT checklist (see link list at top right) with your submission. See author guidelines, under 'Reporting Guidelines'. Please confirm you have submitted this list. | - |
| 17. For tumor marker prognostic studies, we recommend that you follow the REMARK reporting guidelines (see link list at top right). See author guidelines, under 'Reporting Guidelines'. Please confirm you have followed these guidelines. | - |

## F- Data Accessibility

| | |
|---|---|
| 18: Provide a "Data Availability" section at the end of the Materials & Methods, listing the accession codes for data generated in this study and deposited in a public database (e.g. RNA-Seq data: Gene Expression Omnibus GSE39462, Proteomics data: PRIDE PXD000208 etc.) Please refer to our author guidelines for 'Data Deposition'.<br><br>Data deposition in a public repository is mandatory for:<br>a. Protein, DNA and RNA sequences<br>b. Macromolecular structures<br>c. Crystallographic data for small molecules<br>d. Functional genomics data<br>e. Proteomics and molecular interactions | Raw gRNA counts of the CRISPR screen performed in the context of this study can be downloaded from github.com/boutroslab/Supp_Imkeller_2021/external_data/CRISPR_screen_gRNA_counts.csv. All other public datasets used in the study can be accessed as described in the methods section and in the corresponding rmarkdown files. |
| 19. Deposition is strongly recommended for any datasets that are central and integral to the study; please consider the journal's data policy. If no structured public repository exists for a given data type, we encourage the provision of datasets in the manuscript as a Supplementary Document (see author guidelines under 'Expanded View' or in unstructured repositories such as Dryad (see link list at top right) or Figshare (see link list at top right). | - |
| 20. Access to human clinical and genomic datasets should be provided with as few restrictions as possible while respecting ethical obligations to the patients and relevant medical and legal issues. If practically possible and compatible with the individual consent agreement used in the study, such data should be deposited in one of the major public access-controlled repositories such as dbGAP (see link list at top right) or EGA (see link list at top right). | - |
| 21. Computational models that are central and integral to a study should be shared without restrictions and provided in a machine-readable form. The relevant accession numbers or links should be provided. When possible, standardized format (SBML, CellML) should be used instead of scripts (e.g. MATLAB). Authors are strongly encouraged to follow the MIRIAM guidelines (see link list at top right) and deposit their model in a public database such as Biomodels (see link list at top right) or JWS Online (see link list at top right). If computer source code is provided with the paper, it should be deposited in a public repository or included in supplementary information. | The code used for analysis and visualization is deposited at github.com/boutroslab/Supp_Imkeller_2021. |

## G- Dual use research of concern

| | |
|---|---|
| 22. Could your study fall under dual use research restrictions? Please check biosecurity documents (see link list at top right) and list of select agents and toxins (APHIS/CDC) (see link list at top right). According to our biosecurity guidelines, provide a statement only if it could. | No. |
